# DECIDE-Lab: A Value-of-Information and POMDP Framework for Diagnostic Laboratory Test Selection

**DOI:** 10.3390/diagnostics16152356

**Published:** 2026-07-27

**Authors:** Corban Allenbrand

**Affiliations:** Quest Diagnostics, 500 Plaza Drive, Secaucus, NJ 07094, USA; corban.z.allenbrand@questdiagnostics.com

**Keywords:** diagnostic testing, clinical utility, laboratory stewardship, sensitivity, specificity, Bayesian decision analysis, sequential testing, diagnostic dominance

## Abstract

**Background/Objectives:** Diagnostic laboratory tests are often judged by sensitivity and specificity, together with related measures of analytic validity. These assist in deciding on which tests are expected to be more accurate or precise, but they do not determine whether a test result improves patient care. A laboratory test has clinical value when it changes a provider’s decision in a way that improves expected outcomes after accounting for uncertainty and patient burden. This work presents DECIDE-Lab (Decision Centered Evaluation of Clinical Information and Dynamic Evidence for Laboratory Testing), an integrative decision-theoretic framework that adapts value-of-information (VOI) and POMDP methods to laboratory test selection to evaluate the value of single tests and serial testing. **Methods:** The framework connects test performance attributes to clinical utility through posterior belief updating and action thresholds. The concept of clinical utility frontiers is presented, which identifies dominated lab tests. DECIDE-Lab allows preference-sensitive and equity-aware evaluation of lab test diagnostic value by incorporating variation in test performance across patient subgroups. **Results:** An illustrative acute coronary syndrome application shows that the value of serial troponin testing concentrates in intermediate-risk patients for whom an additional result can change disposition or treatment. A worked sepsis pathway demonstrates the POMDP mechanics step by step from an initial belief state to the resulting stop-or-continue decision. Additional disease-state applications demonstrate how the framework can support diagnostic stewardship and adaptive ordering. **Conclusions:** The central implication is that diagnostic value should be measured by action-changing utility rather than accuracy alone, while examining how conclusions change with utilities and implementation constraints.

## 1. Introduction

Diagnostic testing is a core element of medical care because it converts uncertainty into clinical action. A laboratory test result may support actions chosen along many clinical pathways including decisions for treatment. The same result may also create harm through unnecessary treatment or treatment delay. For this reason, diagnostic evaluation that solely judges whether a test is accurate or precise only partially addresses the question of the clinical value of the test. Instead, what must be determined is whether the test changes a clinical decision made by the provider in a direction that creates value for the patient.

The language of diagnostic evaluation emphasizes analytic validity, clinical validity, sensitivity, specificity, likelihood ratios, and predictive values. These measures are indispensable and tell clinicians how a test behaves under specified conditions [1,2,3,4]. Yet they do not answer the test ordering question directly. A highly sensitive and specific test may add little value if the patient already lies below a treatment threshold and the result would keep the patient below the threshold. A faster or less expensive test with lower standalone accuracy may add substantial value if it moves the patient across the threshold at which management changes. Diagnostic value is therefore contextual to the clinical scenario, not intrinsic to the test.

This distinction has practical consequences for laboratory medicine. Many laboratory stewardship programs target duplicate orders or low-yield panels. These efforts can reduce waste, but they often lack a general theory of when a result is worth obtaining. A decision-theoretic approach begins with the downstream action. It asks the following questions: What decision is pending? What is the clinician’s current belief about disease? How would each possible result update that belief? Would the updated belief cross a management threshold? Do the expected benefits of that action change exceed the burden and delay of the test?

Medical decision analysis provides the tools to answer these questions. Treatment-threshold models show when acting, testing, or withholding treatment is rational under uncertainty [5,6,7,8]. Value-of-information (VOI) theory quantifies the expected improvement in decision quality gained by collecting information before acting [9,10,11,12]. Decision curve analysis and cost-effectiveness methods further emphasize that prediction and diagnosis should be evaluated by their consequences for decisions and outcomes [13,14]. These traditions are highly relevant to laboratory testing, but they are rarely presented as a unified framework for test selection, reflex testing, and serial monitoring.

Laboratory diagnosis also unfolds over time. Providers often order an initial test, observe an imperfect result, decide whether to treat or wait, then order a confirmatory or repeat test. Acute coronary syndrome evaluation, sepsis management, autoimmune workups, prostate cancer evaluation, and tuberculosis diagnosis all follow this sequential pattern. Partially observable Markov decision processes (POMDPs) are well suited to these problems because the true disease state is hidden, observations are imperfect, actions affect future states, and the value of testing depends on accumulated evidence [15,16,17,18].

This article develops DECIDE-Lab (Decision Centered Evaluation of Clinical Information and Dynamic Evidence for Laboratory Testing), a framework that combines VOI and POMDP methods for diagnostic laboratory test selection. The framework is designed for clinicians and laboratory scientists, with broader use by health-system decision makers. It is designed to supplement but not replace clinical judgments.

This work provides contributions that are integrative and translational in nature, rather than purely theoretical with a new general-purpose decision algorithm. Bayesian updating, VOI methods, and POMDPs are established components of medical decision analysis. The novelty derives from how DECIDE-Lab organizes these components into a framework that links test performance to action-changing clinical value. Accordingly, the theoretical propositions presented should be interpreted as laboratory-specific implications of established decision-theoretic principles rather than as new general theorems in Bayesian decision theory or POMDP optimization. A coherent specification of how these decision-theoretic principles can be applied together during the selection, sequencing, and termination of diagnostic laboratory testing is the primary methodological contribution from this work.

The framework makes six domain-specific contributions. First, it connects observation-model parameters to posterior beliefs and clinical action thresholds. Second, it distinguishes standalone test value from conditional and sequential value. Third, it defines a clinical utility frontier that identifies dominated laboratory strategies after burden and delay are considered. Fourth, it formalizes optimal stopping for serial and reflex pathways. Fifth, it allows model inputs to vary across patient groups and clinical settings. Sixth, it translates these elements into a workflow for laboratory stewardship and clinical decision support.

## 2. Methods: The DECIDE-Lab Framework

**Figure 1:**
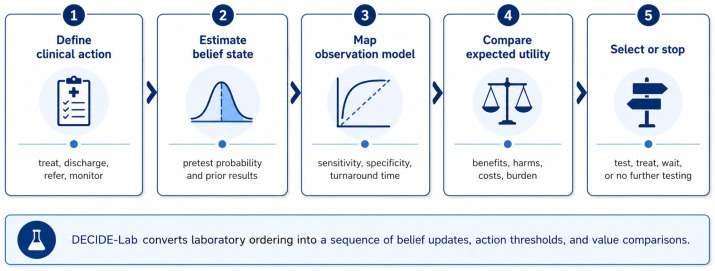
DECIDE-Lab workflow. The framework begins with the clinical action that might change rather than with the laboratory test itself. Test performance enters through the observation model, while clinical value depends on posterior updating and expected utility.

DECIDE-Lab treats laboratory ordering as a decision problem rather than a test-performance problem. The framework has five steps (Figure 1). The provider first defines the pending clinical action. The provider then estimates the current belief state and maps the available tests to observation probabilities. The provider compares the expected utility with and without testing and then acts or continues the diagnostic process.

### 2.1. Disease States, Observations, and Actions

Let D∈{0,1} denote the latent disease state, where D=1 means the disease is present and D=0 means the disease is absent. Let p=P(D=1) denote the provider’s pretest probability. A binary laboratory test *T* produces observation o∈{+,−}. Sensitivity and specificity are

(1) Se=P(T=+∣D=1),Sp=P(T=−∣D=0).

Bayes’ rule gives the posterior probability after a positive result,

(2) $$P\left(D=1|T=+\right)=\frac{Se\;p}{Se\;p+(1-Sp)(1-p)},$$

and after a negative result,

(3) $$P\left(D=1|T=-\right)=\frac{(1-Se)p}{\left(1-Se)p+Sp(1-p\right)}.$$

Let a∈A denote a feasible clinical action. In the simplest case, A={Treat,NoTreat}. In practice, A may include actions such as discharge and treatment, with other pathway-specific choices added as needed. Let U(a,d) denote the utility of action *a* when the true disease state is *d*. Utility may represent net health benefit or net monetary benefit, with other decision-relevant outcomes incorporated when appropriate [6,14].

### 2.2. Generalization Beyond Binary Diseases and Test Results

The binary disease and binary observation formulation is used to provide a transparent introduction to the framework. It is not a structural requirement of DECIDE-Lab. Let the latent clinical state take values in a finite set

(4) $$S=\{s_{1},\ldots ,s_{K}\},$$

where the states may represent competing diagnoses or clinically meaningful disease stages. The belief state is then a probability vector

(5) $$b_{t}=\left(P\left(s_{1}|H_{t}\right),\ldots ,P\left(s_{K}|H_{t}\right)\right),\;\sum_{k=1}^{K}b_{t}\left(s_{k}\right)=1.$$

For a discrete laboratory observation o∈O obtained after test action *a*, the Bayesian update becomes

(6) $$b_{t+1}\left(s_{k}\right)=\frac{Z\left(o|s_{k},a\right)b_{t}\left(s_{k}\right)}{\sum_{j=1}^{K}Z\left(o|s_{j},a\right)b_{t}\left(s_{j}\right)},$$

where $Z(o|s_{k},a)$ is the probability of observation *o* in state $s_{k}$. In this representation, a test can have more than two result categories, such as negative and indeterminate findings.

Continuous biomarkers can be retained on their original measurement scale rather than dichotomized. Let Y∈R denote the observed laboratory value and let $f_{a}\left(y|s_{k}\right)$ denote its state-specific density. The posterior belief after observing Y=y is

(7) $$b_{t+1}\left(s_{k}\right)=\frac{f_{a}\left(y|s_{k}\right)b_{t}\left(s_{k}\right)}{\sum_{j=1}^{K}f_{a}\left(y|s_{j}\right)b_{t}\left(s_{j}\right)}.$$

Expected utility with a continuous observation is obtained by integrating over the possible test values:

(8) $$EU_{a}\left(b\right)=-c_{a}+\int_{Y}p\left(y|b,a\right)\max_{a^{\prime }\in A}\left\{\sum_{k=1}^{K}U\left(a^{\prime },s_{k}\right)b^{\;y,a}\left(s_{k}\right)\right\}\;dy,$$

where $b^{\;y,a}$ is the posterior belief after observing *y*. Numerical quadrature or Monte Carlo integration may be used when a closed-form solution is unavailable. Continuous-observation POMDP methods have been developed for settings in which discretization would discard clinically relevant information [19].

Multiple competing diagnoses can be represented by assigning a state to each diagnostically and therapeutically relevant condition. For example, a patient with acute respiratory symptoms might have states corresponding to bacterial pneumonia or pulmonary embolism, with other causes grouped separately when clinically appropriate. A laboratory observation can increase belief in one state while decreasing belief in several others. The optimal action is selected according to

(9) $$EU_{0}\left(b\right)=\max_{a\in A}\sum_{k=1}^{K}b\left(s_{k}\right)U\left(a,s_{k}\right).$$

In this setting, the scalar treatment threshold used in the binary example is replaced by decision regions within the (K−1)-dimensional belief simplex. Each region contains the beliefs for which a particular clinical action maximizes expected utility. Test value arises when an observation moves the belief vector from one action region to another. The action-changing principle therefore continues to apply, although its geometry is more complex than a single probability threshold.

Disease progression can likewise be represented through a transition matrix or transition model

(10) $$\begin{array}{c} T\left(s_{j}|s_{k},a\right)=P\left(S_{t+1}=s_{j}|S_{t}=s_{k},A_{t}=a\right). \end{array}$$

This allows the framework to distinguish, for example, early sepsis and septic shock while grouping other states as required by the application. The transition probabilities may depend on treatment, elapsed time, and patient characteristics. Although these extensions increase computational burden and may make simple threshold diagrams less intuitive, they remain within the same belief-state and expected-utility formulation.

### 2.3. Expected Value of Diagnostic Information

Without additional testing, the provider selects the action with the highest expected utility:

(11) $$EU_{0}\left(p\right)=\max_{a\in A}\left\{pU\left(a,1\right)+\left(1-p\right)U\left(a,0\right)\right\}.$$

With testing, the provider observes the result before acting:

(12) $$EU_{T}\left(p\right)=-c_{T}+\sum_{o\in \{+,-\}}P\left(o|p,T\right)\max_{a\in A}E\left[U\left(a,D\right)|o\right],$$

where $c_{T}$ includes monetary cost and turnaround time, with other burdens incorporated when relevant. The expected value of diagnostic information is

(13) $$EVDI\left(T;p\right)=EU_{T}\left(p\right)-EU_{0}\left(p\right).$$

Equation (13) shows why accuracy alone is insufficient. Test performance affects posterior probabilities, but utility depends on whether those posterior probabilities change the chosen action. For a treat/no-treat decision, define

(14) $$\begin{array}{cc} U_{TP} & =U(Treat,1) \end{array}$$

(15) $$\begin{array}{cc} U_{FP} & =U(Treat,0) \end{array}$$

(16) $$\begin{array}{cc} U_{FN} & =U(NoTreat,1) \end{array}$$

(17) $$\begin{array}{cc} U_{TN} & =U(NoTreat,0) \end{array}$$

where TP stands for true positive, FP stands for false positive, FN stands for false negative, and TN stands for true negative. Then treatment is preferred when

(18) $$pU_{TP}+\left(1-p\right)U_{FP}\geq pU_{FN}+\left(1-p\right)U_{TN}.$$

Solving gives the treatment threshold

(19) $$p^{*}=\frac{U_{TN}-U_{FP}}{\left(U_{TP}-U_{FN}\right)+\left(U_{TN}-U_{FP}\right)},$$

provided the denominator is positive. This threshold expresses the clinical tradeoff between false-positive and false-negative decisions [5,7].

**Proposition** **1** (Action-Changing Condition)**.**
*A diagnostic test has positive gross value only if at least one possible result changes the action selected under the prior belief. Formally, let A be a finite action set and define the Bayes value function*

(20) $$V\left(p\right)=\max_{a\in A}\left\{pU(a,1)+(1-p)U(a,0)\right\}.$$

*Let O denote the result of a diagnostic test, let $p_{O}=P\left(D=1|O\right)$ be the result-specific posterior, and assume P(O=o)>0 for every result in the support of O. The gross value of the test is*

(21) $$G\left(T;p\right)=E\left[V(p_{O})\right]-V\left(p\right).$$

*Then G(T;p)≥0. Moreover, G(T;p)=0 if there exists an action $a^{*}\in A$ that is optimal at every posterior belief in the support of $p_{O}$. If the posterior support contains beliefs lying in two distinct decision regions and the optimizer is unique within each region, then G(T;p)>0.*

**Proof.**  Suppose that the same action $a^{*}$ maximizes expected utility before testing and after every possible test result. Then the expected utility after observing the test, before subtracting test cost, equals the expected utility of taking $a^{*}$ under the prior by the law of total expectation. Thus the gross value of testing is zero. If testing has a positive cost or burden, the net value is negative. A positive gross value therefore requires at least one result that changes the optimal action. Formally, for each a∈A, the function

(22) $$u_{a}\left(p\right)=pU\left(a,1\right)+\left(1-p\right)U\left(a,0\right)$$

is affine in *p*. Hence $V\left(p\right)=\max_{a\in A}u_{a}\left(p\right)$ is convex as the point-wise maximum of finitely many affine functions. Posterior beliefs satisfy the martingale property

(23) $$E\left[p_{O}\right]=P\left(D=1\right)=p,$$

which follows from the law of iterated expectations. Jensen’s inequality therefore gives

(24) $$E\left[V\left(p_{O}\right)\right]\geq V\left(E\left[p_{O}\right]\right)=V\left(p\right),$$

so G(T;p)≥0.

Now suppose that a common action $a^{*}$ is optimal at every posterior in the support of $p_{O}$. Then

(25) $$\begin{array}{cc} E[V\left(p_{O}\right)] & =E[u_{a^{*}}\left(p_{O}\right)]\\ & =u_{a^{*}}\left(E\left[p_{O}\right]\right)\\ & =u_{a^{*}}\left(p\right)\\ & =V(p), \end{array}$$

because $u_{a^{*}}$ is affine and $a^{*}$ is also optimal at *p*, which is a convex combination of the posterior beliefs. Thus G(T;p)=0.

Finally, suppose the posterior support contains $p_{1}$ and $p_{2}$ with positive probability, where the unique optimal actions differ. Then *V* cannot be affine on the interval joining $p_{1}$ and $p_{2}$; it has a strict kink at a decision boundary between those beliefs. Jensen’s inequality is therefore strict for the nondegenerate posterior distribution, yielding

(26) $$E\left[V\left(p_{O}\right)\right]>V\left(E\left[p_{O}\right]\right)=V\left(p\right).$$

Hence G(T;p)>0.    □

The clinical interpretation is direct in that a test creates value when its result moves the patient’s disease probability and changes the providers clinical decision.

### 2.4. Sensitivity, Specificity, and the Region of Value

Sensitivity and specificity influence value through their effect on posterior beliefs. However, their marginal value is threshold-dependent. A gain in sensitivity has the greatest value when false negatives are harmful and a negative or positive result can change treatment. A gain in specificity has greatest value when false positives are harmful and a result can prevent unnecessary treatment or additional testing.

**Proposition** **2** (Threshold-Dependent Marginal Value)**.** *Improving sensitivity or specificity increases expected utility only through result patterns that can change the optimal action or reduce the expected harm of the action selected. Formally, consider a binary test with sensitivity Se and specificity Sp and a binary action set {Treat,NoTreat}. Assume*

(27) $$U_{TP}>U_{FN}\;\mathrm{and}\;U_{TN}>U_{FP}.$$

*Within any open region of (Se,Sp) in which the optimal result-contingent policy is unchanged, the gross expected utility of testing is affine in (Se,Sp). In particular,*

*1.*  *If the optimal action is the same after positive and negative results, then*
(28) $$\frac{\partial G}{\partial Se}=\frac{\partial G}{\partial Sp}=0.$$
*2.*  *If the optimal policy is to treat after a positive result and not treat after a negative result, then*
(29) $$\frac{\partial G}{\partial Se}=p\left(U_{TP}-U_{FN}\right)>0$$
*and*
(30) $$\frac{\partial G}{\partial Sp}=\left(1-p\right)\left(U_{TN}-U_{FP}\right)>0.$$

*Thus sensitivity and specificity have positive marginal decision value only in regions where test results induce different actions; at boundaries between regions, the value function is continuous but need not be differentiable.*

**Proof.**  Expected utility with testing is a weighted average of posterior optimal utilities across possible observations. Changing sensitivity or specificity changes both observation probabilities and posterior probabilities. If no posterior belief crosses a decision boundary and the same action remains optimal for every observation, the maximized action term remains unchanged except for probability-weighting of the same decision. The expected gain is therefore zero before cost. Positive marginal value arises when the performance change increases the probability of a result that supports a better action or reduces the probability of a result that supports a harmful action. Formally, Fix an open parameter region in which the action selected after each result does not change. If the same action *a* is selected after both results, the gross expected utility is

(31) $$\begin{array}{cc} EU_{T}^{\mathrm{gross}} & =P(O=+)E[U(a,D)|O=+]+P(O=-)E[U(a,D)|O=-]\\ & =E[U(a,D)] \end{array}$$

by the law of total expectation. This expression does not depend on Se or Sp, and therefore both partial derivatives are zero.

Now suppose the fixed contingent policy treats after a positive result and does not treat after a negative result. Conditioning first on the true disease state gives

(32) $$\begin{array}{cc} EU_{T}^{\mathrm{gross}} & =p\left[Se\;U_{TP}+\left(1-Se\right)U_{FN}\right]\\ & +\left(1-p\right)\left[\left(1-Sp\right)U_{FP}+Sp\;U_{TN}\right]. \end{array}$$

Differentiating within the region where this policy remains optimal yields

(33) $$\frac{\partial EU_{T}^{\mathrm{gross}}}{\partial Se}=p\left(U_{TP}-U_{FN}\right)>0$$

and

(34) $$\frac{\partial EU_{T}^{\mathrm{gross}}}{\partial Sp}=\left(1-p\right)\left(U_{TN}-U_{FP}\right)>0.$$

Because $EU_{0}\left(p\right)$ does not depend on Se or Sp, these derivatives are also the derivatives of gross diagnostic value *G*. At a decision boundary, the maximizing contingent policy changes, so *G* remains the maximum of affine functions and is therefore continuous and piecewise affine, but it may fail to be differentiable.    □

This proposition explains why the same test can be of high value in one patient and of low value in another. The key determinant is the patient’s disease probability location relative to clinical decision thresholds.

### 2.5. Handling Utility Elicitation in Clinical Practice

DECIDE-Lab depends on utilities because laboratory tests create value only through the actions that follow them. This requirement is a strength of the framework, but it also creates a practical challenge. Different stakeholders, such as clinicians and patients, may assign different importance to missed diagnoses or unnecessary treatment. DECIDE-Lab makes these judgments and their disagreement visible for analysis.

Utility values can be handled in several ways. First, investigators can use a threshold analysis rather than a single best estimate. Threshold analysis varies the harms of false-negative and false-positive decisions across plausible ranges to identify where the preferred clinical action changes [5,6,8]. Second, stakeholders can elicit utilities from multiple perspectives. A patient-centered analysis may weight anxiety and procedural burden differently from a hospital stewardship analysis. Third, the model can use net monetary benefit or net health benefit when cost-effectiveness assumptions are available [14]. Fourth, the analyst can report robust decision regions, i.e., areas in which the preferred test remains unchanged across a broad range of utilities.

**Figure 2:**
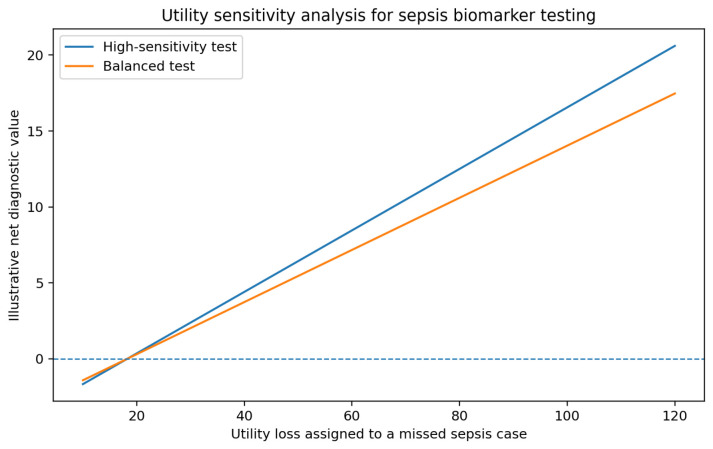
Utility y sensitivity analysis for sepsis biomarker testing. As the utility loss assigned to a missed sepsis case increases, the value of a high-sensitivity testing strategy rises. The purpose is to show how DECIDE-Lab can test robustness when utilities are uncertain or contested. The figure was generated using the Python, version 3.14, script provided in Supplementary Materials S1.

A sepsis biomarker example illustrates the point. In early suspected sepsis, the false-negative consequence may be severe because delayed antibiotics can worsen outcomes. When the utility loss assigned to a missed sepsis case increases, the value of a high-sensitivity test rises relative to a more balanced test, even if the high-sensitivity test produces more false positives. Figure 2 shows this pattern using stylized parameters. The figure is not intended to set a clinical policy. It demonstrates how DECIDE-Lab can reveal whether a recommendation depends on a fragile utility assumption or remains stable across plausible values.

This approach changes how the framework should be interpreted. DECIDE-Lab does not require universally correct utilities. It requires transparent reporting of the utility ranges under which a testing strategy is preferred. That transparency is especially important when diagnostic recommendations affect different stakeholders differently.

### 2.6. Sequential Testing and POMDP Model

Many laboratory pathways involve more than one test. Let $b_{t}=P\left(D=1|H_{t}\right)$ denote the belief state after history $H_{t}$, where the history includes prior laboratory results and treatment response, together with other clinically relevant evidence. If the provider chooses testing action $a_{t}$ and observes $o_{t+1}$, the belief update is

(35) $$b_{t+1}=\frac{P\left(o_{t+1}|D=1,a_{t}\right)b_{t}}{P(o_{t+1}|b_{t},a_{t})}.$$

A diagnostic pathway can be represented as a POMDP

(36) M=(S,A,O,T,Z,R,γ),

where *S* and *A* denote the state and action spaces. The terms *O* and Z(o∣s,a) define the observations and their probabilities, while $T(s^{\prime }|s,a)$ and R(s,a) define transitions and rewards. The term γ is the discount factor. For a laboratory test, sensitivity and specificity parameterize Z(o∣s,a). The discount factor γ models the time value of diagnostic information whereby more timely diagnoses are preferred. Two clinical interpretations of γ are recommended. First, in time-sensitive conditions like sepsis or acute coronary syndrome, delays can yield worse outcomes for patients. A γ<1 ensures rewards achieved sooner are valued more highly than rewards achieved later. For example, a rapid test that leads to immediate correct treatment produces greater reward than a slow test that leads to a correct but delayed reward. Second, a sequence of diagnostic tests may be terminated due to patients failing to follow-up to the first test. A γ<1 introduces a (1−γ>0) probability that diagnostic process prematurely ends.

The optimal value function over belief states satisfies

(37) $$V^{*}\left(b\right)=\max_{a\in A}\left[R\left(b,a\right)+\gamma \sum_{o}P\left(o|b,a\right)V^{*}\left(\tau \left(b,a,o\right)\right)\right],$$

where τ(b,a,o) is the Bayesian belief update after action *a* and observation *o*. After the Bayesian update, R(b,a)=E[R(s,a)|b]=∑sR(s,a)b(s) is the expectation of R(s,a) under the new updated posterior belief.

**Proposition** **3** (Optimal Stopping)**.** *Additional testing is optimal only when its expected marginal value exceeds its total cost, including burden and delay. Formally, let $A_{S}$ denote the set of terminal, non-testing actions and $A_{T}$ the set of available tests. At belief state b, define*

(38) $$V_{stop}\left(b\right)=\max_{a\in A_{S}}R\left(b,a\right)$$

*and, for each test $t\in A_{T}$ with total acquisition cost $c_{t}\left(b\right)$,*

(39) $$Q_{t}\left(b\right)=-c_{t}\left(b\right)+\gamma \sum_{o}P\left(o|b,t\right)V^{*}\left(\tau \left(b,t,o\right)\right).$$

*So the stopping of testing is optimal if and only if*

(40) $$V_{stop}\left(b\right)\geq \max_{t\in A_{T}}Q_{t}\left(b\right).$$

*Equivalently, test t is strictly preferred to stopping if and only if its conditional marginal value*

(41) $$MV_{t}\left(b\right)=\gamma \sum_{o}P\left(o|b,t\right)V^{*}\left(\tau \left(b,t,o\right)\right)-V_{stop}\left(b\right)$$

*strictly exceeds $c_{t}\left(b\right)$.*

**Proof.**  At a belief state $b_{t}$, the provider can stop and choose the best non-testing action or continue testing. Bellman optimality selects the action with the highest expected value. Continuing is preferred only if the expected future value after the next observation, minus the total cost of obtaining that observation, exceeds the value of stopping. Otherwise, stopping dominates. Formally, partition the action set into terminal actions and testing actions. The Bellman equation can then be written as

(42) $$V^{*}\left(b\right)=\max\left\{V_{stop}\left(b\right),\max_{t\in A_{T}}Q_{t}\left(b\right)\right\}.$$

By the definition of the maximum, a terminal action is optimal exactly when its best attainable value is at least as large as the value of every testing action, which is equivalent to

(43) $$V_{stop}\left(b\right)\geq \max_{t\in A_{T}}Q_{t}\left(b\right).$$

For a fixed test *t*, the inequality $Q_{t}\left(b\right)>V_{stop}\left(b\right)$ is equivalent, after rearrangement, to

(44) $$\gamma \sum_{o}P\left(o|b,t\right)V^{*}\left(\tau \left(b,t,o\right)\right)-V_{stop}\left(b\right)>c_{t}\left(b\right),$$

that is, $MV_{t}\left(b\right)>c_{t}\left(b\right)$. Equality produces indifference, and the reverse inequality implies that stopping weakly dominates test *t*.    □

This result gives a formal basis for diagnostic stewardship. Stopping is optimal when further lab test information is unlikely to change the clinical action and does not justify its cost.

### 2.7. Worked Sequential POMDP Walkthrough

A toy example helps clarify how the POMDP component operates in practice. For simplicity, the reward function implicitly enters through the treatment threshold, where above and below the threshold, certain actions are known to yield certain rewards. Consider an adult patient in the emergency department with suspected infection but without definitive evidence of sepsis. The pending decision is whether to start broad-spectrum antibiotics or continue short-interval observation, with further testing treated as an information-generating action.

Let the latent state be S∈{NoSepsis,Sepsis}. Let the initial belief be $b_{0}=P\left(Sepsis\right)=0.25$ after clinical assessment and routine laboratory information. The available actions are two terminal actions, Treat and Observe, plus the information-generating actions $Test_{1}$ and $Test_{2}$. $Test_{1}$ is a rapid screening biomarker with sensitivity $Se_{1}=0.85$ and specificity $Sp_{1}=0.70$. $Test_{2}$ is a more specific follow-up biomarker or confirmatory assessment with sensitivity $Se_{2}=0.75$ and specificity $Sp_{2}=0.90$. The model uses a treatment threshold of 0.50: above this value, immediate treatment has higher expected utility than observation; below this value, observation is preferred unless additional information has positive conditional value.

The first decision is to order $Test_{1}$ because the initial belief lies in the action-changing region. A positive result updates the belief to

(45) $$P\left(Sepsis|Test_{1}+\right)=\frac{0.85(0.25)}{0.85(0.25)+(1-0.70)(0.75)}=0.486.$$

This posterior remains close to the treatment threshold. The patient is neither clearly low risk nor clearly above the threshold for immediate action. Therefore, the conditional value of $Test_{2}$ is evaluated.

If $Test_{2}$ is ordered after a positive $Test_{1}$, the probability of a positive $Test_{2}$ is

(46) $$P\left(Test_{2}+|b=0.486\right)=0.75\left(0.486\right)+\left(1-0.90\right)\left(1-0.486\right)=0.416.$$

A positive $Test_{2}$ increases the posterior to

(47) $$P\left(Sepsis|Test_{1}+,Test_{2}+\right)=\frac{0.75(0.486)}{0.75(0.486)+0.10(0.514)}=0.876,$$

which favors treatment. A negative $Test_{2}$ lowers the posterior to

(48) $$P\left(Sepsis|Test_{1}+,Test_{2}-\right)=\frac{0.25(0.486)}{0.25(0.486)+0.90(0.514)}=0.208,$$

which favors observation or an alternative diagnostic pathway. Thus, $Test_{2}$ has conditional value after a positive $Test_{1}$ because either result changes the preferred action.

A negative $Test_{1}$ leads to a different conclusion. The posterior becomes

(49) $$P\left(Sepsis|Test_{1}-\right)=\frac{\left(1-0.85)(0.25\right)}{\left(1-0.85)(0.25)+0.70(0.75\right)}=0.067.$$

At this belief state, the patient is well below the treatment threshold. Unless the clinician assigns an extremely high utility loss to a missed case or observes new clinical deterioration, the conditional value of $Test_{2}$ is low. The POMDP therefore recommends stopping the biomarker sequence and choosing observation or reassessment.

**Figure 3:**
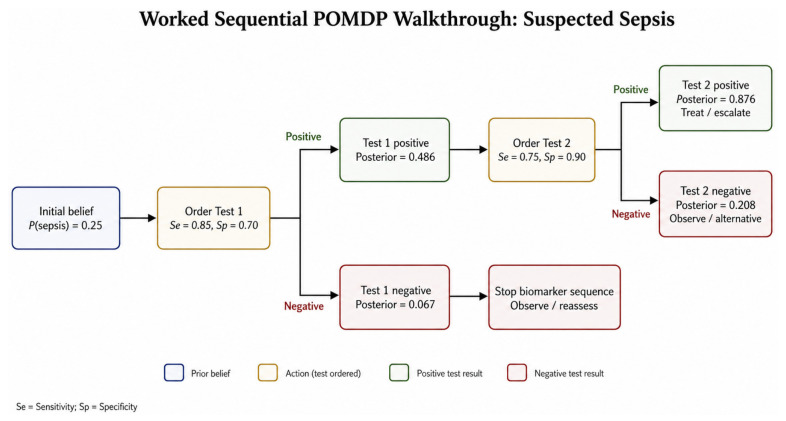
Worked POMDP walkthrough for suspected sepsis. The pathway begins with an initial belief state of 0.25. A positive first test leaves the patient near the treatment threshold, so the conditional value of a second test is high. A negative first test lowers the belief state enough that the model stops the biomarker sequence. The figure was generated using the Python, version 3.14, script provided in Supplementary Materials S1.

Table 1 summarizes the belief trajectory and stop-or-continue logic. Figure 3 provides the same logic as a pathway diagram. The important lesson is that the POMDP does not recommend serial testing automatically. It recommends the next test only when the current belief state makes the next observation likely to change the clinical action.

### 2.8. Clinically Grounded Sepsis Case Study

The preceding walkthrough was intentionally stylized to demonstrate the mechanics of sequential belief updating. This section provides a more clinically grounded example using representative values from the sepsis literature and applies the single-stage DECIDE-Lab calculations in Equations (11)–(13), followed by a finite-horizon sequential-testing analysis. The purpose is to demonstrate model implementation rather than establish a clinical sepsis protocol. Sepsis is a heterogeneous clinical syndrome defined by life-threatening organ dysfunction caused by a dysregulated host response to infection, and no individual laboratory biomarker independently establishes or excludes the diagnosis [20,21]. Biomarker testing should not delay guideline-directed treatment when sepsis or septic shock is sufficiently likely [22]. All calculations were performed using unrounded probabilities; displayed probabilities and utility values are rounded to three and two decimal places, respectively.

#### 2.8.1. Clinical Case and Parameterization

Consider a 71-year-old patient presenting to the emergency department with suspected community-acquired pneumonia, a temperature of 39.1 °C, heart rate of 118 beats/min, respiratory rate of 28 breaths/min, mean arterial pressure of 63 mmHg, leukocyte count of $18.2\times 10^{9}$/L, and new organ dysfunction corresponding to an estimated increase in the Sequential Organ Failure Assessment score of three points. Blood cultures, supportive care, and other time-critical interventions proceed according to institutional protocols. The specific decision modeled here is whether to initiate antimicrobial treatment or continue short-interval observation while pursuing an alternative diagnostic pathway.

Let D=1 denote bacterial sepsis for which immediate antimicrobial treatment is beneficial and D=0 denote the absence of that state. The terminal actions are

(50) A={Treat,Observe}.

After integrating the suspected infectious source and organ dysfunction, together with the remaining clinical information, suppose that a locally calibrated risk model estimates the initial probability as

(51) $$p_{0}=P\left(D=1\right)=0.30.$$

Two laboratory observations are evaluated. Procalcitonin (PCT) is modeled as an adjunctive marker of bacterial infection using representative pooled sensitivity and specificity estimates of 0.77 and 0.79 [23]. Lactate is primarily a marker of physiological stress and illness severity rather than a specific diagnostic test for sepsis. It is included as an illustrative rapid, lower-cost, but less discriminating observation. The utility values and total test costs are shown in Table 2. Test cost is expressed in utility units and incorporates direct cost, turnaround time, treatment delay, patient burden, and downstream resource use.

Using Equation (19), the utilities imply a treatment threshold of

(52) $$\begin{array}{cc} p^{*} & =\frac{U_{TN}-U_{FP}}{\left(U_{TP}-U_{FN}\right)+\left(U_{TN}-U_{FP}\right)}\\ & =\frac{100-45}{\left(100-0)+(100-45\right)}=0.355. \end{array}$$

Thus, treatment is preferred when the posterior probability is at least 0.355. Because $p_{0}=0.30$, observation is initially preferred, but an informative positive result may change the action.

#### 2.8.2. No-Test Decision and Bayesian Updating

Following Equation (11), the expected utilities of treatment and observation before additional testing are

(53) $$\begin{array}{cc} EU(\mathrm{Treat}|p_{0}) & =0.30(100)+0.70(45)=61.50, \end{array}$$

(54) $$\begin{array}{cc} EU(\mathrm{Observe}|p_{0}) & =0.30(0)+0.70(100)=70.00. \end{array}$$

Therefore,

(55) $$EU_{0}\left(p_{0}\right)=\max\left\{61.50,70.00\right\}=70.00,$$

and observation is the optimal no-test action.

For PCT, the probability of a positive result is

(56) P(PCT+)=0.77(0.30)+0.21(0.70)=0.378.

Applying Equation (2), the posterior after a positive result is

(57) $$P\left(D=1|\mathrm{PCT}+\right)=\frac{0.77(0.30)}{0.77(0.30)+0.21(0.70)}=0.611.$$

The remaining posteriors are calculated analogously and are summarized in Table 3. Both tests are action-changing in the base case because a positive result raises the posterior above $p^{*}=0.355$, whereas a negative result leaves it below the threshold.

#### 2.8.3. Expected Utility and Diagnostic Value

The posterior expected utilities after a positive PCT result are

(58) $$\begin{array}{cc} EU(\mathrm{Treat}|\mathrm{PCT}+) & =0.611(100)+0.389(45)=78.61, \end{array}$$

(59) $$\begin{array}{cc} EU(\mathrm{Observe}|\mathrm{PCT}+) & =0.611(0)+0.389(100)=38.89. \end{array}$$

Treatment is therefore optimal after a positive result. After a negative result, observation is optimal with an expected utility of 88.91. Applying Equation (12),

(60) $$\begin{array}{cc} EU_{\mathrm{PCT}}\left(p_{0}\right) & =-c_{\mathrm{PCT}}+P\left(\mathrm{PCT}+\right)\max_{a}E\left[U\left(a,D\right)|\mathrm{PCT}+\right]\\ & +P\left(\mathrm{PCT}-\right)\max_{a}E\left[U\left(a,D\right)|\mathrm{PCT}-\right]\\ & =-3.00+0.378\left(78.61\right)+0.622\left(88.91\right)\\ & =82.02. \end{array}$$

The resulting expected value of diagnostic information is

(61) $$EVDI(\mathrm{PCT};p_{0})=82.02-70.00=12.02.$$

Repeating the same calculation for lactate gives

(62) $$EU_{L}\left(p_{0}\right)=73.19,\;EVDI\left(L;p_{0}\right)=3.19.$$

As shown in Table 4, PCT provides greater value in the base case because its positive and negative results produce greater separation around the treatment threshold. The conclusion is nevertheless conditional on the prior, test performance, utilities, and total test costs rather than being an intrinsic property of PCT.

#### 2.8.4. Conditional Value and Optimal Stopping

DECIDE-Lab also evaluates whether a second test should be obtained after the first result. At an updated belief state $b_{1}$, define the conditional value of a candidate second test as

(63) $$CV_{T_{2}}\left(b_{1}\right)=EU_{T_{2}}\left(b_{1}\right)-EU_{0}\left(b_{1}\right).$$

Continuing is optimal only when this quantity is positive. This is the finite-horizon stop-or-continue comparison represented by the Bellman equation in Equation (37) and Proposition 3.

Consider first a PCT-first strategy. After a positive PCT result, $b_{1}=0.611$. A subsequent positive or negative lactate result produces posteriors of 0.748 and 0.520, respectively. Both remain above the treatment threshold, so treatment remains optimal regardless of the lactate result. Lactate therefore has zero gross action-changing value, and its conditional net value equals its negative cost:

(64) $$CV_{L}\left(0.611\right)=-1.50.$$

After a negative PCT result, $b_{1}=0.111$. The corresponding post-lactate beliefs are 0.190 and 0.079, both of which remain below the treatment threshold. Thus,

(65) $$CV_{L}\left(0.111\right)=-1.50.$$

The optimal PCT-first policy therefore stops after either result:

(66) $$\pi_{\mathrm{PCT}-\mathrm{first}}^{*}=\left\{\begin{array}{cc} \mathrm{Treat}\;\mathrm{and}\;\mathrm{stop}, & \mathrm{if}\;\mathrm{PCT}+;\\ \mathrm{Observe}\;\mathrm{and}\;\mathrm{stop}, & \mathrm{if}\;\mathrm{PCT}-. \end{array}\right.$$

The reverse sequence yields a different result. After a positive lactate result, $b_{1}=0.447$, and PCT has conditional value

(67) $$CV_{\mathrm{PCT}}\left(0.447\right)=80.33-69.57=10.76.$$

After a negative lactate result, $b_{1}=0.228$, and

(68) $$CV_{\mathrm{PCT}}\left(0.228\right)=82.84-77.20=5.64.$$

PCT therefore has positive conditional value after either lactate result. The expected utility of the complete lactate-first sequence is

(69) $$\begin{array}{cc} EU_{L\to \mathrm{PCT}} & =-1.50+0.329(80.33)+0.671(82.84)\\ & =80.52. \end{array}$$

Although this sequential strategy is better than no testing, it is inferior to PCT alone:

(70) $$EU_{\mathrm{PCT}}-EU_{L\to \mathrm{PCT}}=82.02-80.52=1.50.$$

The sequential result illustrates two central properties of DECIDE-Lab. First, the availability of a second test does not imply that the test should be ordered. After PCT, lactate cannot change the action and has negative conditional value. Second, a lower-cost initial test may be dominated when a more informative test is subsequently required on every branch. As summarized in Table 5, the optimal base-case policy is to order PCT directly, treat after a positive result, observe after a negative result, and stop the biomarker sequence.

#### 2.8.5. Sensitivity Analysis

The base-case recommendation was evaluated against changes in the prior probability and utility weights, with test characteristics varied in a separate analysis. A summary of selected sensitivity analysis results are given in Table 6. The first analysis varies the prior probability while retaining the base-case treatment threshold. Before accounting for test cost, PCT can change the treatment decision across an approximate prior interval of 0.13 to 0.65. The corresponding interval for the illustrative lactate observation is approximately 0.23 to 0.45. Net-positive-value intervals are narrower because the expected benefit must also exceed the test burden. These intervals confirm that testing is most useful at intermediate belief states. At very low probabilities, even a positive result may not justify treatment; at very high probabilities, even a negative result may not justify withholding treatment.

The second analysis varies the utility of missed or delayed sepsis, $U_{FN}$, while retaining the other utilities. The resulting treatment threshold is

(71) $$p^{*}\left(U_{FN}\right)=\frac{55}{155-U_{FN}}.$$

Increasing the penalty for missed sepsis lowers the treatment threshold and may shift the optimal strategy toward immediate treatment rather than additional testing. Thus, assigning a severe consequence to a false-negative decision does not necessarily imply that more testing is optimal; it may imply that waiting for information is itself undesirable. Conversely, increasing the burden assigned to unnecessary treatment raises the threshold and increases the value of specific confirmatory evidence.

The maximum justifiable test cost equals the gross expected value of information. In the base case, PCT retains positive net value while its total cost remains below approximately 15.02 utility units. The corresponding break-even cost for lactate is approximately 4.69 units. These costs include turnaround delay as well as direct price. In time-sensitive presentations, a slower but more accurate test may lose value if waiting for its result reduces the expected benefit of correct treatment.

Sensitivity and specificity may also vary by assay threshold and patient comorbidity, with additional heterogeneity assessed when supported by data. For subgroup *g*, DECIDE-Lab replaces pooled performance estimates with

(72) $$Se_{T,g}=P\left(T+|D=1,g\right),\;Sp_{T,g}=P\left(T-|D=0,g\right),$$

and recalculates subgroup-specific posterior probabilities and expected utilities. When subgroup estimates are imprecise, probabilistic sensitivity analysis should be used instead of unstable point estimates.

The sequential calculations assume conditional independence between PCT and lactate given the latent disease state. If the biomarkers remain correlated after conditioning on disease, the incremental value of the second test may be overstated. A clinical implementation should therefore estimate the joint observation model $P(T_{2}|D,T_{1})$ from patient-level data whenever sequential testing is being evaluated.

#### 2.8.6. Implementation Requirements and Limitations

Practical implementation requires a locally calibrated prior-risk model and assay-specific performance estimates. It also requires explicit clinical actions and locally appropriate operational inputs. These inputs could be incorporated into a web-based calculator or electronic health record decision-support service. The clinician-facing output should report the current belief and action threshold. It should also show the recommended strategy and its sensitivity to uncertain inputs.

The case study has several limitations. It uses a binary disease state and two terminal actions, although sepsis is dynamic and may require multiple latent states and actions. The utility weights and lactate performance parameters are illustrative. Pooled PCT estimates may not generalize across age groups or clinical settings. The initial prior may also be uncertain or change rapidly during deterioration. Operational constraints such as test availability and prolonged turnaround time may further alter the preferred policy.

Accordingly, the base-case result should not be interpreted as a universal recommendation to order PCT in suspected sepsis. The example demonstrates a reproducible decision process: estimate the current belief, identify the optimal action without testing, calculate result-specific posterior actions, compare expected utility with and without testing, order a second test only when its conditional value is positive, and stop when additional information cannot improve the clinical decision. Local calibration and prospective validation would be required before clinical deployment.

### 2.9. Clinical Utility Frontier and Diagnostic Dominance

A laboratory test can be dominated even when it has favorable test-performance characteristics. Define the expected utility of test $T_{i}$ at belief state *b* as $EU(T_{i};b)$ and its total cost as $C(T_{i};b)$. Test $T_{i}$ is weakly dominated by $T_{j}$ if

(73) $$EU\left(T_{j};b\right)\geq EU\left(T_{i};b\right),\;C\left(T_{j};b\right)\leq C\left(T_{i};b\right),$$

with at least one strict inequality. The clinical utility frontier is the set of non-dominated testing strategies across relevant belief states.

**Proposition** **4** (Diagnostic Dominance)**.** *A dominated test should not be selected by a rational provider unless it has benefits not captured in the base utility function, such as availability or patient preference. Formally, fix a belief state b and two feasible tests $T_{i}$ and $T_{j}$. Let G(T;b) denote gross expected utility after observing test T and acting optimally, and let C(T;b) denote its total cost. Define net value by*

(74) N(T;b)=G(T;b)−C(T;b).

*If*

(75) $$G\left(T_{j};b\right)\geq G\left(T_{i};b\right)\;\mathrm{and}\;C\left(T_{j};b\right)\leq C\left(T_{i};b\right),$$

*with at least one strict inequality, then*

(76) $$N\left(T_{j};b\right)>N\left(T_{i};b\right).$$

*Consequently, $T_{i}$ cannot be optimal among the feasible tests under the specified model.*

**Proof.**  If one test produces at least as much expected utility at no greater cost, and one inequality is strict, then the dominated test cannot maximize expected net value under the specified utility function. Selection of the dominated test can only be justified by adding omitted benefits or constraints to the model. Formally, subtracting net values gives

(77) $$\begin{array}{cc} N\left(T_{j};b\right)-N\left(T_{i};b\right) & =\left[G\left(T_{j};b\right)-G\left(T_{i};b\right)\right]+\left[C\left(T_{i};b\right)-C\left(T_{j};b\right)\right]. \end{array}$$

Each bracketed term is nonnegative by assumption, and at least one is strictly positive. Therefore,

(78) $$N\left(T_{j};b\right)-N\left(T_{i};b\right)>0,$$

which implies $N\left(T_{j};b\right)>N\left(T_{i};b\right)$. Hence $T_{i}$ cannot maximize net value over any feasible choice set that also contains $T_{j}$. If $T_{i}$ is nevertheless selected, at least one relevant attribute, constraint, or preference must be absent from the stated functions *G* and *C* or from the feasible set.    □

The frontier concept is useful for diagnostic development and laboratory stewardship. It identifies redundant tests, tests that add value only after particular prior results, and rapid tests that dominate slower alternatives when delay carries clinical risk.

### 2.10. Preference-Sensitive and Equity-Aware Diagnostic Value

Diagnostic value can vary across patients and populations. Patients may differ in risk tolerance, ability to return for follow-up, out-of-pocket cost, transportation burden, fear of procedures, treatment preferences, and willingness to accept false-positive or false-negative risk. Subgroups may also differ in disease prevalence, disease spectrum, access to confirmatory care, and test performance [2,4]. A uniform testing threshold can therefore appear fair while producing unequal value.

Let *g* index a patient subgroup or preference profile. Subgroup-specific diagnostic value is

(79) $$EVDI_{g}\left(T;b\right)=EU_{g}\left(T;b\right)-EU_{g}\left(NoTest;b\right).$$

Equation (79) allows priors and utilities to vary by subgroup, with other inputs adapted when supported by evidence. A subgroup-specific threshold is not automatically equitable. It becomes equity-relevant when it is used to examine whether a uniform policy underdiagnoses one group or imposes burdens unequally.

Prostate cancer screening and diagnostic follow-up illustrate the issue. A uniform PSA threshold for MRI referral or biopsy may treat equal PSA values as equal clinical situations. Yet the decision value of follow-up can differ when baseline risk, probability of clinically significant cancer, MRI availability, biopsy access, complication risk, and loss to follow-up vary across groups [24,25]. If one subgroup has a higher prior probability of clinically significant disease and worse access to timely MRI or biopsy, a uniform threshold may delay definitive evaluation for patients who face both higher disease risk and greater follow-up barriers. Conversely, if another subgroup has lower prior risk and reliable access to follow-up, the same threshold may expose more patients to false-positive cascades without comparable benefit.

DECIDE-Lab can evaluate this problem explicitly. The analyst defines subgroup-specific priors $p_{g}$ and test characteristics $Se_{g}$ and $Sp_{g}$, then incorporates follow-up and utility differences when supported by evidence. The utility function may emphasize cancer detection and biopsy harm. The framework then compares a uniform rule with subgroup-specific strategies that maximize expected utility subject to an equity constraint. One possible constraint is that the probability of missed clinically significant cancer should not exceed a prespecified level in any subgroup. Another is that expected net benefit should not fall below a minimum acceptable level for disadvantaged groups.

**Figure 4:**
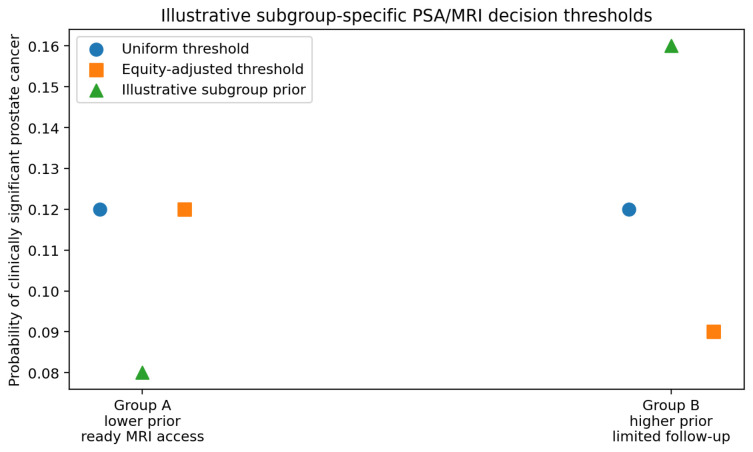
Equity-aware prostate cancer example. A uniform PSA/MRI threshold may not produce equal decision value when subgroup prior risk and access to follow-up differ. DECIDE-Lab can compare uniform and subgroup-specific strategies under explicit fairness constraints. The figure was generated using the Python, version 3.14, script provided in Supplementary Materials S1.

Figure 4 shows a stylized example. The uniform threshold treats the groups identically. The equity-adjusted threshold differs because the model accounts for subgroup-specific prior risk and follow-up constraints. This does not imply that demographic group membership should be used uncritically in clinical algorithms. It shows how a decision model can expose the consequences of ignoring heterogeneity. The ethical question is not whether thresholds differ but whether the resulting policy improves fair outcomes without reinforcing bias or denying beneficial care.

The equity contribution of DECIDE-Lab is therefore diagnostic rather than prescriptive. It identifies where equal rules produce unequal expected consequences. It also separates empirical questions from normative questions. Empirical questions concern prevalence and test performance. Normative questions concern utility maximization and equal opportunity for diagnosis. Publication of both sets of assumptions would make diagnostic stewardship more transparent.

### 2.11. Learning DECIDE-Lab Inputs from Clinical Data

The preceding formulation treats the prior, observation model, transition model, and utilities as known inputs. In practice, these quantities can be estimated from electronic health records and prospective diagnostic cohorts. DECIDE-Lab therefore separates two related tasks: statistical learning estimates the uncertain clinical environment, while decision analysis uses those estimates to select an action.

The patient-specific prior or belief state can be estimated with a calibrated predictive model

(80) $${\hat{b}}_{t}=g_{\theta }\left(X_{0:t}\right),$$

where $X_{0:t}$ may include vital signs and prior laboratory values, together with other available clinical data. Depending on the application, $g_{\theta }$ may be a regression model or a temporal neural network. Large-scale EHR models can use longitudinal and multimodal information, but their output must be calibrated if it is to be interpreted as a belief state rather than only as a ranking score [26,27].

The observation model can be estimated from patients for whom both the laboratory result and an appropriate reference outcome are available. For discrete results, the model estimates

(81) $$\hat{Z}\left(o|s,a,x\right)=P\left(O=o|S=s,A=a,X=x\right),$$

which permits sensitivity, specificity, and indeterminate-result probabilities to vary with patient characteristics *x*. For continuous tests, the model estimates the conditional density $f_{a}\left(y|s,x\right)$. Flexible regression or machine-learning models can therefore replace fixed population-average test characteristics when sufficient data are available.

Longitudinal data can be used to estimate the transition model

(82) $$\hat{T}\left(s^{\prime }|s,a,x,\Delta t\right)=P\left(S_{t+\Delta t}=s^{\prime }|S_{t}=s,A_{t}=a,X_{t}=x\right).$$

Multistate survival models or hidden Markov models may be appropriate, depending on how the latent states and observation times are defined. The transition model should account for treatment because clinical actions may alter subsequent disease progression.

Utilities require a different type of estimation. Observed outcomes and costs can inform some utility estimation. However, the utility of an action cannot always be identified from observed associations because treatment selection is confounded by clinical severity. Estimation of action consequences may therefore require randomized evidence or target-trial emulation. Patient preferences and treatment burden may additionally require direct elicitation rather than prediction from routine EHR data.

After these components are estimated, DECIDE-Lab performs policy optimization using the learned model. This modular structure permits a range of predictive algorithms to supply inputs without allowing the prediction model itself to determine the action. A model with higher discrimination is not necessarily associated with greater decision value if it is poorly calibrated near an action threshold or if its errors occur in patients for whom management would change.

Model development should therefore evaluate calibration and clinical decision performance in addition to discrimination. External validation is needed because prevalence and clinical workflows may differ across institutions. Calibration can also change after deployment, including within clinically important subgroups, requiring monitoring and recalibration [28].

An alternative is to learn the policy more directly from observational data using offline reinforcement learning or related policy-learning methods. Such methods are attractive when the number of possible histories is large, but they introduce substantial identification and validation challenges. The observed data contain outcomes primarily for actions selected by the historical clinical policy. Evaluation of a new policy therefore requires off-policy evaluation and adequate overlap between historical and proposed actions. Unmeasured confounding and distribution shift can produce unsafe recommendations [29,30].

## 3. Results

### 3.1. Quantitative Clinical Application

The quantitative application considers suspected acute coronary syndrome and serial troponin testing. The examples are stylized and intended to demonstrate the decision logic rather than estimate a definitive clinical policy. Acute coronary syndrome is useful because the clinical decision is time-sensitive, the disease state is partially observed, and repeat testing is common [31,32,33].

**Figure 5:**
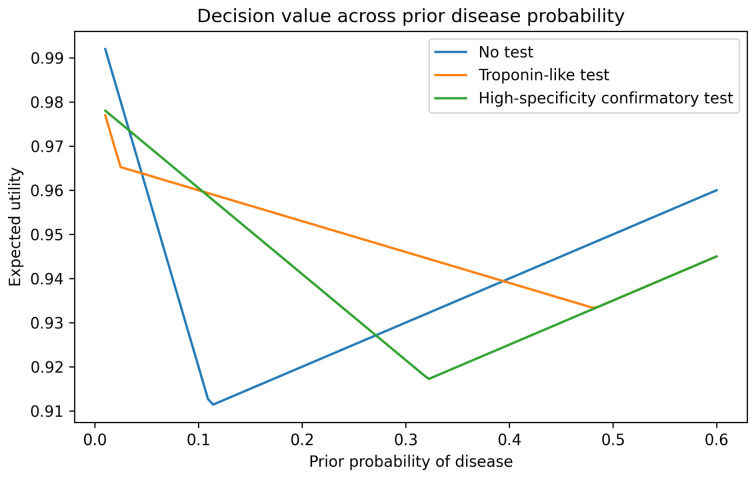
Illustrative relationship between pretest probability and expected value of diagnostic information. Test value concentrates in the intermediate-risk region, where results are most likely to change action. Values are stylized and generated from the Supplementary Materials S1. The figure was generated using the Python, version 3.14, script provided in Supplementary Materials S1.

The provider begins with a pretest probability of myocardial infarction. The main actions are discharge and observation, with escalation added when clinically indicated. The principal harms are missed myocardial infarction and unnecessary admission. The model compares no further testing with single-stage and sequential troponin strategies. Figure 5 shows how diagnostic value varies with the prior probability of disease.

The main result is qualitative but important. Testing has low value when the pretest probability is very low because discharge or no acute treatment is already preferred. Testing also has low value when the pretest probability is very high because treatment or admission is already preferred. Testing has the greatest value in the intermediate range, where either a positive or negative result can change disposition or treatment. This is the action-changing region predicted by Proposition 1.

**Figure 6:**
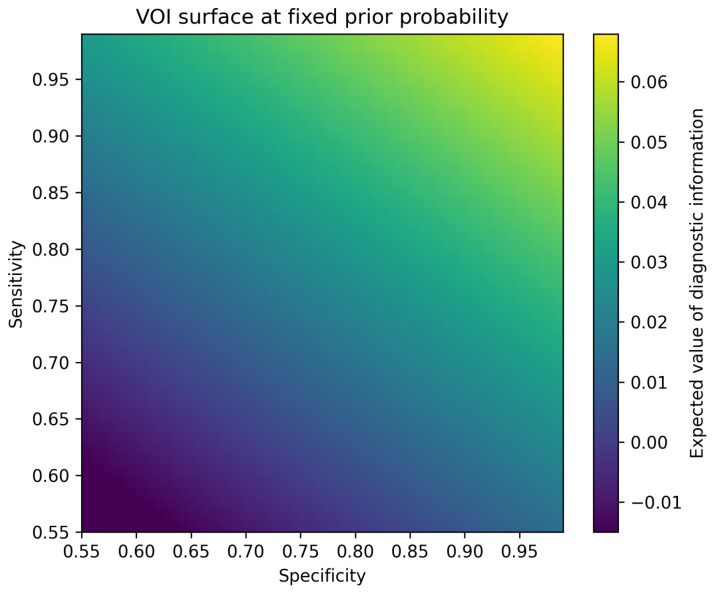
VOI surface for diagnostic testing as a function of prior probability and test performance. The surface highlights that high accuracy does not guarantee high value when posterior beliefs do not cross action thresholds. The figure was generated using the Python, version 3.14, script provided in Supplementary Materials S1.

Figure 6 illustrates how value changes jointly with prevalence and test performance. Improvements in sensitivity and specificity matter most when they alter action around a clinically relevant threshold. This pattern supports the claim that test performance should be interpreted through decision value rather than accuracy alone.

### 3.2. Qualitative Clinical Applications

The five applications in this section show how DECIDE-Lab can guide real diagnostic pathways. Each application follows the same structure: define the pending decision and belief state, then compare test-guided actions by expected utility. Testing stops when another result is unlikely to change management. Table 7 summarizes the decision points and test sequences for the five applications and can be used for a high-level overview of how the DECIDE-Lab framework can be adapted to diverse and important diagnostic challenges.

#### 3.2.1. Acute Coronary Syndrome and Serial Troponin Testing Decisions

In suspected acute coronary syndrome, the immediate decision is not simply whether myocardial infarction is present. The provider must decide whether to discharge or continue observation, with escalation considered when indicated. DECIDE-Lab estimates the belief state from the initial troponin and electrocardiographic findings.

A single troponin result has value when it moves the posterior probability across a discharge, observation, admission, or treatment threshold. A repeat troponin has value only if the patient remains in an intermediate belief state after the first result. Thus, the framework can compare 0/1 h, 0/2 h, and 0/3 h strategies by asking which patients remain in the action-changing region after each observation [31,32,33].

The framework can also clarify why faster tests may be valuable even when their sensitivity and specificity are similar to slower alternatives. If a rapid result shortens observation time and reduces unnecessary admission, speed enters the utility function. In that setting, the rapid test may lie on the clinical utility frontier even if another strategy has slightly better analytic performance.

#### 3.2.2. Sepsis Biomarkers and Antibiotic Decisions

Sepsis illustrates dynamic diagnostic value because the latent state can worsen quickly and the harms of delay are asymmetric. The provider must decide whether to start antibiotics or continue diagnostic clarification, with escalation handled by the broader care pathway. The belief state includes vital signs and organ dysfunction.

DECIDE-Lab separates two uses of biomarkers. Early in evaluation, lactate or procalcitonin may help decide whether to escalate care or start antibiotics. Later, repeated procalcitonin may help decide whether to discontinue antibiotics and reduce avoidable exposure [34]. These decisions have different thresholds. The same test can therefore have low value for initiating antibiotics in a high-risk patient but high value for stopping antibiotics after clinical stabilization.

A POMDP representation is particularly useful because sepsis is a transition process. States may distinguish localized infection from septic shock, with other stages added when clinically necessary. Actions affect future outcomes, and observations arrive over time. The model can evaluate whether new biomarker information justifies its delay and cost.

#### 3.2.3. Prostate Cancer Testing and Biopsy Decisions

Prostate cancer evaluation involves repeated decisions under uncertainty. The provider must decide whether to repeat PSA, order reflex biomarkers, obtain MRI, refer to urology, perform biopsy, continue surveillance, or treat. The relevant disease state is often not just any prostate cancer but clinically significant prostate cancer. The belief state may include age, family history, race, PSA level, PSA velocity, digital rectal examination, prior biopsy, and patient preferences [24,25].

The framework identifies where each test is most useful. PSA has screening value but limited specificity. MRI may have high decision value in intermediate-risk patients because it can reduce unnecessary biopsy while preserving detection of clinically significant disease. Biopsy may dominate when the posterior probability already exceeds the biopsy threshold. Conversely, additional imaging may have little value in very low-risk patients because surveillance remains preferred regardless of result.

The POMDP extension supports longitudinal surveillance. Repeated PSA values and MRI findings update the belief state. Utilities should emphasize cancer detection and biopsy complications. This application shows why targeted diagnostic sequencing can be more valuable than uniform testing.

#### 3.2.4. Autoimmune Disease Testing Decisions

Autoimmune disease evaluation often creates cascades of laboratory testing. A positive antinuclear antibody test can trigger anti-dsDNA testing or complement measurement, with further evaluation guided by clinical findings. DECIDE-Lab asks whether each step changes referral or treatment enough to justify its cost.

The prior belief should reflect clinical features, organ involvement, medication exposure, family history, baseline laboratory abnormalities, and symptom evolution. ANA testing may have value in patients with compatible symptoms and intermediate pretest probability. It may have low value in patients with nonspecific symptoms and very low pretest probability because false-positive follow-up may dominate benefit. Specific antibody panels may have high conditional value after a positive ANA in a clinically compatible patient but low value after an isolated low-titer ANA in an otherwise low-risk patient [35].

A sequential model helps prevent indiscriminate reflex testing. The next test should be selected only if it can change referral or treatment decisions. The framework therefore supports more transparent autoimmune test stewardship.

#### 3.2.5. Tuberculosis Testing Decisions

Tuberculosis diagnosis differs by clinical setting, prevalence, exposure history, immune status, and public health consequences. The provider may need to decide whether to treat latent infection, isolate a patient, start empiric therapy, order nucleic acid amplification testing, send culture, or begin contact investigation. The belief state includes symptoms, exposure risk, country of origin, immune suppression, radiographic findings, and local prevalence [36,37].

DECIDE-Lab clarifies why test value changes across settings. In a low-prevalence outpatient setting, specificity and avoidance of unnecessary treatment may dominate. In a high-risk inpatient setting with possible active tuberculosis, sensitivity and speed may dominate. A rapid molecular test may create high value by shortening the time to isolation or treatment even when culture remains necessary for confirmation and susceptibility testing.

A POMDP can distinguish latent infection from active disease, with additional states introduced when needed. Rewards may emphasize patient outcomes and transmission prevention. This application shows that diagnostic value can include both individual and population-level consequences.

## 4. Discussion

DECIDE-Lab reframes diagnostic laboratory testing as decision-centered information acquisition. This reframing is the main contribution of the article. It explains why laboratory tests should not be evaluated only by sensitivity and specificity. Those quantities are essential inputs, but the clinical endpoint is improved action. Across clinical applications, DECIDE-Lab provides a practical workflow. First, define the pending decision and estimate the current belief state. Next, compare expected utility with and without testing. If uncertainty remains, evaluate the next test and stop when additional information no longer justifies its burden.

The framework also helps interpret apparent conflicts between accuracy and value. A test with excellent discrimination may have low value if the provider’s action would not change. A test with modest discrimination may have high value if it moves an intermediate-risk patient across a decision threshold. A rapid test may dominate a slower test when delay carries clinical harm. A confirmatory test may have high value only after a particular first result. These distinctions are difficult to express with accuracy metrics alone.

For laboratories, DECIDE-Lab provides a formal basis for reflex testing and stewardship rules. A reflex test should be ordered when conditional value remains positive after the preceding result. It should be suppressed when the previous result already places the patient below or above an action threshold. For clinicians, the framework provides a language for deciding when to stop testing. For health systems, it distinguishes tests that improve expected utility from those that add cost without changing management.

The clinical utility frontier adds a useful comparison tool. A test may be dominated if another strategy delivers equal or greater expected utility at equal or lower cost. This frontier can compare individual tests and reflex algorithms. It can also support diagnostic development by identifying whether a new assay must improve speed or sensitivity to create incremental value.

The preference-sensitive and equity-aware extension broadens the framework. Diagnostic value depends not only on biology and accuracy but also on patient preferences and follow-up access. A single testing policy may not create equal value for all patients. Formal subgroup-specific VOI can help reveal when policies improve equity and when they reproduce inequity. The prostate cancer example illustrates why this matters: a uniform PSA-related threshold can produce different access and harm across groups.

DECIDE-Lab is a framework contribution: it defines a reusable problem representation, identifies the inputs needed for laboratory applications, and makes explicit the relationships between diagnostic accuracy and posterior belief, then connects them to optimal stopping. Its purpose is to improve the formulation and evaluation of laboratory-ordering decisions rather than to replace general POMDP solvers or existing decision-theoretic algorithms. The framework’s added value is therefore primarily found at the intersection of these established methods. Standard evaluations often consider test discrimination and treatment thresholds separately. DECIDE-Lab treats these as linked components of one diagnostic pathway. This integration makes it possible to identify situations in which an accurate test is clinically redundant, a less accurate but faster test is preferred, a reflex test has value only after a particular preceding result, or additional testing should stop because no remaining observation can justify its burden. The intended contribution for operations and AI planning communities is a domain formulation that translates laboratory medicine into a structured information-acquisition and stopping problem. For laboratory medicine and clinical informatics, the contribution is a unified method for moving from test-performance metrics to patient- and pathway-specific decision value.

### 4.1. Comparison with Alternative Approaches to Diagnostic Test Evaluation

DECIDE-Lab builds on several established approaches to medical decision-making but addresses a more specific question. Traditional diagnostic accuracy studies evaluate how well a test distinguishes patients with and without a target condition using measures such as sensitivity and specificity. These measures are essential for characterizing the observation model, but they do not directly establish whether a test result changes a clinical action or whether the expected benefit of that change exceeds the test’s cost, burden, and delay. DECIDE-Lab extends diagnostic performance analysis by linking test results to posterior beliefs, action thresholds, and expected clinical utility.

Treatment-threshold analysis is closely related to DECIDE-Lab because it identifies the disease probability at which treatment becomes preferable to withholding treatment. DECIDE-Lab incorporates this logic but extends it from the question of whether to act to the additional questions of which laboratory test should be ordered, whether a subsequent test has conditional value, and when further testing should stop. This distinction is particularly important in reflex and serial testing pathways, where the value of a second test depends on the posterior belief created by the first result.

Decision curve analysis evaluates the net benefit of using a prediction model or diagnostic strategy across a range of threshold probabilities. It is particularly useful for determining whether a model-guided strategy offers greater clinical value than default approaches such as treating all or treating none. DECIDE-Lab is complementary to decision curve analysis but differs in its emphasis on adaptive decision making. Decision curve analysis commonly evaluates a fixed model or policy at a single decision point, whereas DECIDE-Lab updates beliefs after each laboratory observation and can optimize a sequence of testing and treatment decisions. Decision curve analysis could therefore be used to evaluate a fixed DECIDE-Lab policy externally, while DECIDE-Lab itself determines the adaptive policy to be evaluated.

Conventional decision-tree analysis can represent test results and clinical actions, together with their expected consequences. For short diagnostic pathways with a small number of predefined branches, a decision tree may be transparent and sufficient. However, decision trees become increasingly cumbersome when testing is repeated, when the next test depends on the complete history of prior observations, or when stopping decisions must be reconsidered at multiple stages. DECIDE-Lab reduces this complexity by representing the information relevant to the next decision through the posterior belief state. This permits repeated Bayesian updating and a formal comparison between stopping and continuing without requiring every possible clinical history to be treated as an unrelated branch.

Cost-effectiveness analysis addresses whether the incremental health benefits of an intervention or strategy justify its incremental costs, often using quality-adjusted life-years, lifetime costs, and incremental cost-effectiveness ratios. Its primary perspective is commonly that of a population, payer, or health system. DECIDE-Lab addresses the more proximal clinical question of which test, if any, should be ordered next for a patient at a particular belief state. The two approaches are complementary rather than competing. Cost-effectiveness analysis can provide long-term costs and health outcomes that enter the DECIDE-Lab utility function. DECIDE-Lab then uses those values to determine whether acquiring additional diagnostic information improves the current decision and whether a later test remains worthwhile after earlier results are known.

Markov state-transition models are useful for representing disease progression and long-term outcomes. However, conventional Markov models do not necessarily distinguish between the true clinical state and the imperfect observations available to the clinician. The POMDP component of DECIDE-Lab makes this distinction explicit. The disease state may remain hidden, laboratory results provide imperfect observations, and actions may affect both future outcomes and the information available at later decision points. This feature is particularly relevant in acute coronary syndrome, sepsis, longitudinal cancer surveillance, and other diagnostic settings in which both the patient’s condition and the available evidence evolve over time.

Value-of-information analysis provides the direct conceptual foundation for DECIDE-Lab by quantifying the expected improvement in decision quality from obtaining additional information before acting. DECIDE-Lab specializes this principle for laboratory medicine by connecting sensitivity and specificity to posterior beliefs, action thresholds, test burden, turnaround time, and downstream clinical consequences. It also extends single-stage value-of-information analysis to conditional and sequential value. A test may have positive value at the beginning of a pathway but no value after an earlier result has moved the patient away from an action threshold. Conversely, a test with modest standalone value may have high conditional value when an earlier result leaves the patient in a narrow region of residual uncertainty.

DECIDE-Lab’s principal advantage is therefore greatest in diagnostic pathways that are adaptive rather than fixed. These include serial testing and time-sensitive pathways in which a test’s value depends on previous results. The framework is also useful when the same laboratory test may be valuable for one patient but redundant for another because their prior probabilities lie in different regions relative to a treatment or disposition threshold. By incorporating turnaround time and patient burden, DECIDE-Lab can also identify when a faster test has greater value than a delayed alternative.

The clinically grounded sepsis example illustrates these distinctions. A conventional diagnostic accuracy comparison identifies PCT as the more discriminating of the two modeled observations. A cost-effectiveness analysis could compare the average costs and outcomes of PCT-guided and lactate-guided strategies across a patient population. DECIDE-Lab additionally identifies why PCT has positive value at the patient’s current belief state, why lactate has no conditional value after either PCT result, and why a lactate-first strategy is dominated when PCT must subsequently be ordered on both branches. The framework therefore answers not only whether testing is beneficial on average, but also which test should be ordered next, whether another result can still change management, and when the diagnostic sequence should terminate.

DECIDE-Lab may offer less incremental advantage when the diagnostic problem consists of a single test followed by a single irreversible action, the disease state is effectively static, turnaround time is negligible, and no adaptive follow-up testing is contemplated. In such settings, a conventional decision tree or treatment-threshold analysis may be sufficient and easier to implement. The additional complexity of DECIDE-Lab is most justified when partial observability, sequential evidence, dynamic disease progression, or optimal stopping materially affects the clinical decision.

### 4.2. Path to Clinical Decision Support Implementation

The practical path to implementation begins with narrow use cases rather than a comprehensive diagnostic optimizer. A health system could first deploy DECIDE-Lab for a single pathway with clear actions and measurable outcomes, such as serial troponin testing or procalcitonin-guided antibiotic discontinuation. The model would sit behind an electronic health record clinical decision support rule. At the time of ordering, the system would retrieve recent laboratory results and the pending order. It would then estimate the current belief state and return a concise recommendation based on whether the proposed test could change management.

The recommendation should be designed for clinical workflow. A useful display would not show the full POMDP. It would show the current risk category and the reason for the recommendation. For example, the system might state that repeat testing is low value because the prior result already places the patient below the discharge threshold, or that repeat testing remains useful because the patient is still in the intermediate-risk region. This explanation is important for clinician trust and for avoiding alert fatigue.

Computational burden depends on the size of the state and action spaces. Small binary or few-state models can be evaluated in real time with precomputed threshold tables. Many laboratory stewardship applications do not require online solution of a large POMDP. A practical implementation can pre-solve the model offline across a grid of priors, test results, time intervals, and utility values. The EHR tool then performs a table lookup or a small Bayesian update at the bedside. Larger models can use approximate dynamic programming, finite-horizon truncation, sparse action spaces, or policy rules learned from simulation [16,17,18].

DECIDE-Lab is the computational engine for modeling diagnostic pathways. A bedside “clinical calculator” requires EHR integration and local calibration. Local inputs should include institutional prevalence and turnaround time. Laboratory leaders and clinicians should review local prevalence estimates and recommended thresholds as part of a governed path to implementation. After deployment, the system should be monitored for ordering behavior and missed diagnoses.

The calculator need not rely exclusively on manually specified risk estimates. A calibrated prediction service can transform current EHR data into the initial belief state and update that belief as new observations become available. Separate models can estimate patient-specific test performance or disease transitions. The decision-support layer would then pass these estimates to the DECIDE-Lab value function rather than converting a machine-learning score directly into an order recommendation.

This separation between prediction and decision is intentional. The predictive model estimates what is likely to be true, whereas DECIDE-Lab determines what information or action has the greatest expected value. Each layer requires separate validation. The prediction layer requires calibration and monitoring for dataset shift. The decision layer requires validation of utilities and policy value.

### 4.3. Limitations

This study has several limitations that are important when translating DECIDE-Lab from a methodological framework into clinical practice. The quantitative applications are illustrative and were not calibrated to a single patient-level clinical cohort. The numerical results therefore demonstrate the decision logic of DECIDE-Lab rather than define validated testing policies. Before deployment, each application would require local calibration and external validation. It would also require prospective evaluation and continued monitoring for unintended clinical or operational effects.

#### 4.3.1. Uncertainty in Parameters During Acute and Critical Illness

DECIDE-Lab requires an estimate of the current disease probability. However, the pretest probability may be especially uncertain during acute and critical presentations. This problem is particularly relevant in shock and sepsis, as well as other rapidly evolving conditions. Clinical findings may be incomplete when the first decision must be made. Observations may also arrive at different times, while the underlying disease state changes during the diagnostic process. Furthermore, prevalence estimates derived from historical cohorts may not accurately represent either the individual patient or the current clinical environment.

The posterior trajectory and optimal policy depend on the initial belief. As a result, even a modest error in the estimated prior can move a patient across an action threshold. This may change the recommended test sequence. It may also alter the treatment decision or the point at which testing stops.

This limitation can be addressed by treating the prior as uncertain and dynamic rather than as a fixed, precisely known input. Analysts should evaluate recommendations over a plausible interval or probability distribution of priors. They should also report whether the preferred policy remains stable across that range. The belief state should be updated whenever new clinical findings become available or when the patient’s physiological condition changes. Treatment response may also provide information that warrants a revised belief state.

When nearby prior values produce different optimal actions, the model should communicate that the recommendation is sensitive to risk estimation. It should not present a single deterministic result as though the underlying risk were known with certainty. In rapidly evolving illness, elapsed time and state transitions should also be modeled explicitly. A policy developed for a stable patient may no longer be appropriate after clinical deterioration.

#### 4.3.2. Clinical Resources and Operational Constraints

The theoretical formulation assumes that tests and clinical actions can be selected from a defined feasible set. In practice, the available options may be constrained by laboratory operating hours or assay availability. Reagent shortages and analyzer downtime may further limit access. Staffing levels and specimen transportation can prolong the time required to obtain a result. Sample-volume requirements or delayed result verification may also make a test impractical. In addition, confirmatory testing may not be available within the relevant clinical time window.

The financial price of a test may be less important than the clinical consequences of waiting for it. Additional phlebotomy may increase patient burden. Delayed results may postpone treatment. Patients may also be lost to follow-up before confirmatory testing is completed. False-positive results can create further downstream testing and resource use. A policy that appears optimal in an unconstrained model may therefore be infeasible or harmful in practice.

Operational feasibility should be incorporated into the model before optimization. A test that is unavailable should be removed from the feasible action set. Uncertain availability can be represented probabilistically. Prolonged turnaround time can be modeled as a direct cost, a transition delay, or a reduction in future reward through the discount factor. The utility function should also reflect patient burden and expected downstream resource use.

Policies should be recalculated when local capacity or turnaround times change. In some settings, a rapid test with lower diagnostic accuracy may provide greater clinical value than a more accurate test whose result arrives too late to influence the decision. DECIDE-Lab should therefore be interpreted as optimizing among clinically feasible actions rather than among all tests that are theoretically available.

#### 4.3.3. Uncertainty and Context Dependence of Utility Parameters

The framework requires explicit utilities for the outcomes that follow clinical decisions. These may include correct treatment and delayed diagnosis, with other consequences incorporated when relevant. Utilities may also reflect anxiety or resource use. In many clinical settings, these values are unavailable or difficult to elicit. They may also be disputed because patients, clinicians, laboratories, payers, and health systems can value the same outcome differently.

Utility values may change across patients and populations. Age and comorbidity can alter the consequences of treatment. Access to follow-up and individual preferences may also change the relative value of competing outcomes. A utility vector calibrated in one population may therefore have limited relevance in another.

DECIDE-Lab should not rely on a single assumed set of utility values without examining their uncertainty. Utility estimates can be informed by stakeholder elicitation and empirical outcome data. Patient-preference studies may provide additional evidence. When appropriate, net health benefit or net monetary benefit can be used to place costs and outcomes on a common scale.

Sensitivity analysis should identify the utility values at which the preferred test or action changes. This analysis may be performed one parameter at a time or across several parameters jointly. Probabilistic sensitivity analysis can be used when the inputs are represented by distributions rather than point estimates. Results should be presented as robust decision regions or as the probability that each strategy is optimal. This is preferable to issuing a recommendation based on one unverified utility vector.

Subgroup-specific utilities may be appropriate when the consequences of testing or treatment differ meaningfully across populations. However, such models require careful validation. Otherwise, they may encode unsupported assumptions or reinforce existing inequities. Without population-specific calibration and transparent reporting of utility assumptions, the clinical relevance of a DECIDE-Lab recommendation is necessarily limited.

#### 4.3.4. Computational Complexity and Clinician Interpretability

POMDPs can become computationally demanding as the model expands. The burden increases when there are many latent states or candidate tests. It also increases when the model includes repeated observations, multiple actions, or long diagnostic horizons. Exact solution methods may become impractical in large or continuous state spaces. Correlated observations and operational constraints add further complexity.

Approximate methods can reduce this computational burden. Examples include point-based value iteration and finite-horizon truncation. These approaches improve tractability, but approximation may introduce error. It may also make the resulting policy more difficult to validate.

The full POMDP computation does not need to be exposed at the bedside. Small diagnostic pathways can often be solved offline over a grid of plausible beliefs and test results. Time intervals and utility values can also be incorporated into this grid. The clinical system may then perform only a Bayesian update or table lookup in real time.

Computational tractability alone is not sufficient for clinical use. A decision-support tool must explain why a test is recommended or why further testing should stop. The interface should display the current estimated risk and action threshold. The main assumptions driving the recommendation should also be visible. The system should indicate when the result is sensitive to uncertain priors, utility values, or test performance.

Interpretability is especially important because a precise recommendation may conceal uncertain inputs or omitted clinical factors. DECIDE-Lab should therefore function as transparent decision support rather than as an autonomous ordering system. Clinicians must retain the ability to override a recommendation and document information that is not represented in the model. They must also be able to recognize when the patient’s condition has changed enough that the prior policy is no longer applicable.

Prospective studies should assess clinician understanding and override behavior. Alert fatigue should also be evaluated explicitly.

#### 4.3.5. Simplified State and Observation Models

The binary disease and binary test formulation used in the principal derivations is a simplifying presentation. Many diagnostic problems involve competing diseases and continuous biomarker trajectories, with further complexity added by disease progression. Although DECIDE-Lab can represent these settings through vector-valued beliefs, multistate transition models, and continuous observation densities, the resulting models require more data and are less easily summarized by a single treatment threshold.

With multiple states, action boundaries become regions in a multidimensional belief space. With continuous biomarkers, expected utility generally requires integration over the observation distribution. Correlated longitudinal measurements may further require a joint temporal observation model. These extensions can improve clinical realism but may reduce interpretability and increase both estimation error and computational burden. The binary examples should therefore be interpreted as transparent special cases that demonstrate the framework’s logic rather than as the only forms supported by the model.

#### 4.3.6. Future Work

These limitations define the empirical requirements for future research. DECIDE-Lab applications should be calibrated with representative electronic health record data and laboratory information system data. They should then be tested in clinically important subgroups and under the operational conditions in which they are intended to function.

Prospective evaluations should compare model-guided policies with existing care. Patient outcomes should be assessed alongside missed diagnoses and unnecessary treatment. Testing burden and turnaround time should also be measured. Additional outcomes should include resource use, equity, and clinician acceptance.

Initial implementation should focus on narrow diagnostic pathways. These pathways should have a limited state and action space. They should also have clear decision thresholds and reliable data inputs. Outcomes must be measurable within a clinically meaningful period. The framework should be extended to larger pathways only after it has demonstrated adequate calibration and clinical benefit in focused settings.

A major direction for future research is the empirical estimation of DECIDE-Lab inputs from longitudinal clinical data. Studies should compare interpretable statistical models with flexible machine-learning approaches for estimating belief states, observation models, and disease transitions. These comparisons should emphasize calibration near clinical action boundaries rather than discrimination alone.

Future studies should also examine whether a prediction-plus-optimization architecture is safer and more transferable than policies learned end-to-end from observational data. Retrospective policy evaluation and controlled implementation studies will be needed before learned DECIDE-Lab policies can be considered suitable for clinical use.

## 5. Conclusions

Laboratory tests create value when they improve clinical decisions in a way that creates value for patients. DECIDE-Lab provides a rigorous and clinically interpretable framework for evaluating that value. By combining VOI with POMDPs, the framework links test characteristics to posterior beliefs and sequential testing while retaining utility and equity considerations. The approach supports a shift from asking whether a test is accurate to asking whether the test changes care in a way that benefits patients. 

## Figures and Tables

**Table 1:** Step-by-step POMDP walkthrough for a stylized suspected sepsis pathway.

Step	Belief State	Action	Result and Posterior	Decision Implication
Initial assessment	$b_{0}=0.25$	Order $Test_{1}$	Not yet observed	Prior is intermediate; information can change action.
After $Test_{1}+$	$b_{1}=0.486$	Evaluate $Test_{2}$	Near treatment threshold	Continue testing because $Test_{2}$ can separate treat from observe.
After $Test_{1}+,Test_{2}+$	$b_{2}=0.876$	Stop testing	Posterior high	Treat or escalate; more biomarker testing has low marginal value.
After $Test_{1}+,Test_{2}-$	$b_{2}=0.208$	Stop testing	Posterior lower	Observe, reassess, or pursue alternative diagnoses.
After $Test_{1}-$	$b_{1}=0.067$	Stop biomarker sequence	Posterior low	Observation or alternative workup preferred unless clinical status changes.

**Table 2:** Base-case parameters for the clinically grounded sepsis analysis. Lactate performance values are illustrative and should not be interpreted as universal diagnostic estimates.

Input	Sensitivity	Specificity	Model Cost	Interpretation
Procalcitonin	0.77	0.79	3.0	Adjunctive evidence regarding bacterial infection.
Lactate threshold	0.49	0.74	1.5	Illustrative rapid physiological-risk observation.
Treat, D=1	$U_{TP}=100$		Correct early treatment	
Treat, D=0	$U_{FP}=45$		Unnecessary antimicrobial treatment	
Observe, D=1	$U_{FN}=0$		Missed or delayed treatment	
Observe, D=0	$U_{TN}=100$		Correct observation	

**Table 3:** Posterior probabilities and optimal actions after each possible laboratory observation.

Observation	Probability	Posterior P(D=1∣o)	Optimal Action
PCT positive	0.378	0.611	Treat
PCT negative	0.622	0.111	Observe
Lactate positive	0.329	0.447	Treat
Lactate negative	0.671	0.228	Observe

**Table 4:** Base-case expected utility and expected value of diagnostic information.

Strategy	Gross Expected Utility	Net Expected Utility	EVDI
No additional testing	70.00	70.00	0.00
Lactate-guided management	74.69	73.19	3.19
PCT-guided management	85.02	82.02	12.02

**Table 5:** Conditional-value and optimal-stopping calculations for the two possible test sequences.

First Result	Stop Value	Value with Second Test	Optimal Decision
PCT positive	78.61	77.11	Treat and stop
PCT negative	88.91	87.41	Observe and stop
Lactate positive	69.57	80.33	Order PCT
Lactate negative	77.20	82.84	Order PCT

**Table 6:** Selected sensitivity-analysis results.

Parameter	Value or Boundary	Decision Implication
PCT action-changing prior interval	0.13–0.65	Potential value is concentrated at intermediate pretest probabilities.
Lactate action-changing prior interval	0.23–0.45	The less discriminating observation is useful across a narrower risk interval.
$U_{FN}=0$	$p^{*}$ = 0.355	A severe false-negative penalty produces a relatively low treatment threshold.
$U_{FN}=40$	$p^{*}$ = 0.478	Reducing the relative penalty for missed sepsis raises the threshold and can increase the role of confirmatory testing.
PCT break-even test cost	15.02 units	PCT loses positive net value above this total modeled cost.
Lactate break-even test cost	4.69 units	The lower-information test tolerates a smaller total burden.

**Table 7:** Structured comparison of clinical applications across the key dimensions of the framework.

Application	Acute Coronary Syndrome (ACS)	Sepsis	Prostate Cancer	Autoimmune Disease	Tuberculosis (TB)
Decision Point	Admit, discharge, or observe/re-test	Initiate antibiotics, obtain cultures, or observe	Biopsy, MRI, or active surveillance	Refer to rheumatology, order specific panel, or monitor	Treat latent TB, start empiric therapy, or isolate
Belief State (Priors)	P(MI) based on symptoms, ECG, initial troponin, risk factors	P(Sepsis) based on vital signs, lactate, clinical signs of infection	P(Cancer) based on PSA, DRE, age, family history	P(Disease) based on symptoms, organ involvement, ANA result	P(TB) based on exposure, symptoms, immune status, IGRA/TST
Action Threshold	Probability thresholds for discharge vs. observation vs. admission	Probability threshold for initiating empiric antibiotics	Probability threshold for recommending MRI or biopsy	Probability threshold for specialty referral or advanced testing	Probability thresholds for treatment vs. isolation
Test Sequence	Serial troponin	Procalcitonin, lactate, blood cultures	PSA → Reflex markers → mpMRI → Biopsy	ANA → dsDNA panels → Complement levels	TST/IGRA → Chest X-ray → Nucleic acid test → Culture
Utility Considerations	Harm of missed MI or harm of unnecessary admission	Harm of delayed antibiotics, or antibiotic overuse	Harm of missed cancer or overdiagnosis	Harm of delayed diagnosis or cost of test cascades	Harm of missed diagnosis or drug toxicity

## Data Availability

The original contributions presented in this study are included in the article/Supplementary Materials. Further inquiries can be directed to the corresponding author.

## Supplementary Materials

The following supporting information can be downloaded at https://www.mdpi.com/article/10.3390/diagnostics16152356/s1, Supplementary Materials S1: DECIDE-Lab: A Value-of-Information and POMDP Framework for Diagnostic Laboratory Test Selection.
