# Supplementary material for: DECIDE-Lab: A Value-of-Information and POMDP Framework for Diagnostic Laboratory Test Selection
Source: Diagnostics (Basel). 2026 Jul 27;16(15):2356. doi: 10.3390/diagnostics16152356 (PMC13464772; doi:10.3390/diagnostics16152356)
Supplement: Supplementary file 1 [file diagnostics-16-02356-s001.zip › diagnostics-4392979-supplementary.pdf]

Supplementary Material S1

DECIDE-Lab: A Value-of-Information and POMDP Framework for Diagnostic Laboratory Test Selection

Purpose of this Supplement

This appendix gives python code to reproduce the figures presented in the manuscript. Its purpose is to ensure complete reproducibility of every figure appearing in the manuscript. The parameter values used throughout this appendix are illustrative and are intended to demonstrate the proposed decision-theoretic framework rather than provide disease-specific clinical recommendations.

Requirements and Python Version

The analyses were performed using Python 3.12 and the following libraries

- NumPy version 2.4.6
- Pandasv version 2.3.3
- Matplotlib version 3.11

Required packages may be installed using

```
pip install numpy pandas matplotlib
```

Reproducing the Manuscript

Executing

```
python generate_figures.py
```

produces the following directory with figures and supporting data files (.csv).

```
figures/

    voi_prior_sweep.png

    voi_surface.png

    sepsis_utility_sensitivity.png

    equity_psa_thresholds.png
```

`pomdp_sepsis_walkthrough.png`

`diagnostic_voi_prior_sweep.csv`

`sepsis_utility_sensitivity.csv`

`sepsis_pomdp_walkthrough.csv`

Description of Manuscript Figures

Figure 2: Utility Sensitivity Analysis

Purpose was to demonstrate how prevalence, downstream access to care, and clinical utility can justify subgroup-specific testing thresholds while maintaining transparent decision rules. It illustrated Recommendation 1 discussed in the manuscript by evaluating how the value of a high-sensitivity sepsis biomarker changes as the utility loss associated with missed sepsis increases. Generated by

```
make_utility_and_equity_figures()
```

Figure 3. Sequential POMDP Walkthrough

Purpose was to provide a worked example illustrating sequential laboratory testing for suspected sepsis.Generated by

```
make_sepsis_pomdp_walkthrough()
```

The workflow illustrates

- Initial belief state
- Bayesian updating after Test 1
- Conditional value of ordering Test 2
- Updated posterior belief
- Optimal stopping decisions

Figure 4: Equity-Aware Diagnostic Thresholds

Purpose was to llustrates subgroup-specific diagnostic thresholds for the prostate cancer example discussed in the manuscript and to demonstrat how prevalence, downstream access to care, and clinical utility can justify subgroup-specific testing thresholds while maintaining transparent decision rules. Generated by

```
make_utility_and_equity_figures()
```

Figure 5: Value of Information Across Prior Disease Probability

Purpose was to illlustrates how expected utility varies across prior disease probabilities for multiple diagnostic strategies.
Generated by

```
make_diagnostic_voi_figures()
```

Outputs

- Expected utility curves
- Decision regions
- Prior probability sensitivity

Figure 6:Value-of-Information Surface

Purpose was to illustrate the joint influence of sensitivity and specificity on diagnostic value.
Generated by

```
make_diagnostic_voi_figures()
```

Outputs

- Expected value of information
- Sensitivity-specificity tradeoffs
- Heat map visualization

Mathematical Details

Posterior probabilities are computed using Bayes' theorem.
Positive test

$$P(D|+) = \frac{SeP(D)}{SeP(D) + (1 - Sp)(1 - P(D))}$$

Negative test

$$P(D|-) = \frac{(1 - Se)P(D)}{(1 - Se)P(D) + Sp(1 - P(D))}$$

Expected value of diagnostic information

$$VOI = E[U|Test] - E[U|NoTest]$$

Sequential testing

$$VOI_2 = E[U|Test_2, Test_1] - E[U|Stop]$$

Additional testing is recommended whenever

$$VOI_2 > 0.$$

Illustrative Simulation Parameters

Table 1: Illustrative parameter values used throughout the computational examples.

Example	Parameter	Value
Sepsis	Prior probability	0.25
Sepsis	Test 1 sensitivity	0.85
Sepsis	Test 1 specificity	0.70
Sepsis	Test 2 sensitivity	0.75
Sepsis	Test 2 specificity	0.90
Utility analysis	False-positive penalty	4
Utility analysis	Testing cost	2.5
Equity example	Subgroup priors	0.08, 0.16

These values are intended solely to illustrate the computational framework.

Output Files

Running the Python script produces

- diagnostic_voi_prior_sweep.csv
- sepsis_utility_sensitivity.csv
- sepsis_pomdp_walkthrough.csv

These files contain the numerical values underlying the manuscript figures.

Code Availability

The Python code used to generate the figures is provided below.
The code contains

- Bayesian updating
- Expected utility calculations

- Value-of-information calculations
- Sequential POMDP illustration
- Utility sensitivity analysis
- Equity-aware threshold analysis
- Figure generation

Supplementary Code S1

The complete Python implementation is reproduced below.

```python
from pathlib import Path
import numpy as np
import pandas as pd
import matplotlib.pyplot as plt
from matplotlib.patches import FancyBboxPatch, FancyArrowPatch

OUT = Path(".")
FIGDIR = OUT / "figures"
FIGDIR.mkdir(exist_ok=True)

def post_pos(p, se, sp):
    """Posterior probability after a positive binary test result."""
    return se * p / (se * p + (1 - sp) * (1 - p))


def post_neg(p, se, sp):
    """Posterior probability after a negative binary test result."""
    return (1 - se) * p / ((1 - se) * p + sp * (1 - p))

def make_diagnostic_voi_figures():
    U_TP = 1.0
    U_TN = 1.0
    U_FP = 0.90
    U_FN = 0.20
    TEST_COST = 0.015

    def treat_utility(p):
        return p * U_TP + (1 - p) * U_FP

    def notreat_utility(p):
        return p * U_FN + (1 - p) * U_TN

    def best_utility(p):
        return max(treat_utility(p), notreat_utility(p))
```

```

def test_utility(p, se, sp, cost=TEST_COST):
    pp = se * p + (1 - sp) * (1 - p)
    pn = 1 - pp
    return -cost + pp * best_utility(post_pos(p, se, sp)) + pn *
        best_utility(post_neg(p, se, sp))

priors = np.linspace(0.01, 0.60, 120)
strategies = {
    "No test": [best_utility(p) for p in priors],
    "Troponin-like test": [test_utility(p, 0.89, 0.82) for p in
       priors],
    "High-specificity confirmatory test": [test_utility(p, 0.75,
        0.95) for p in priors],
}

df = pd.DataFrame({"prior_probability": priors, **strategies})
df.to_csv(OUT / "diagnostic_voi_prior_sweep.csv", index=False)

plt.figure(figsize=(7, 4.5))
for name in strategies:
    plt.plot(priors, strategies[name], label=name)
plt.xlabel("Prior probability of disease")
plt.ylabel("Expected utility")
plt.title("Decision value across prior disease probability")
plt.legend()
plt.tight_layout()
plt.savefig(FIGDIR / "voi_prior_sweep.png", dpi=300)
plt.close()

se_grid = np.linspace(0.55, 0.99, 80)
sp_grid = np.linspace(0.55, 0.99, 80)
P = 0.15
voi = np.zeros((len(se_grid), len(sp_grid)))
base = best_utility(P)

for i, se in enumerate(se_grid):
    for j, sp in enumerate(sp_grid):
        voi[i, j] = test_utility(P, se, sp) - base

plt.figure(figsize=(6, 5))
plt.imshow(
    voi,
    origin="lower",
    aspect="auto",
    extent=[sp_grid.min(), sp_grid.max(), se_grid.min(), se_grid
       .max()],
```

```python
    )
    plt.colorbar(label="Expected value of diagnostic information")
    plt.xlabel("Specificity")
    plt.ylabel("Sensitivity")
    plt.title("VOI surface at fixed prior probability")
    plt.tight_layout()
    plt.savefig(FIGDIR / "voi_surface.png", dpi=300)
    plt.close()

def make_utility_and_equity_figures():
    missed_harms = np.linspace(10, 120, 80)
    prior, cost, fp_harm = 0.22, 2.5, 4.0

    def diagnostic_value(se, sp, missed_harm):
        return prior * se * missed_harm - (1 - prior) * (1 - sp) * fp_harm - cost

    hi = [diagnostic_value(0.92, 0.62, h) for h in missed_harms]
    bal = [diagnostic_value(0.78, 0.80, h) for h in missed_harms]

    pd.DataFrame(
        {
            "missed_sepsis_harm": missed_harms,
            "value_high_sensitivity": hi,
            "value_balanced": bal,
        }
    ).to_csv(OUT / "sepsis_utility_sensitivity.csv", index=False)

    plt.figure(figsize=(7.5, 4.8))
    plt.plot(missed_harms, hi, label="High-sensitivity test")
    plt.plot(missed_harms, bal, label="Balanced test")
    plt.axhline(0, linestyle="--", linewidth=1)
    plt.xlabel("Utility loss assigned to a missed sepsis case")
    plt.ylabel("Illustrative net diagnostic value")
    plt.title("Utility sensitivity analysis for sepsis biomarker testing")
    plt.legend()
    plt.tight_layout()
    plt.savefig(FIGDIR / "sepsis_utility_sensitivity.png", dpi=300)
    plt.close()

    subgroups = [
        "Group A\nlower prior\nready MRI access",
        "Group B\nhigher prior\nlimited follow-up",
    ]
    prior_vals = [0.08, 0.16]
    thresholds = [0.12, 0.09]
```

```python
    uniform = [0.12, 0.12]
    x = np.arange(len(subgroups))

    plt.figure(figsize=(7.5, 4.6))
    plt.scatter(x - 0.08, uniform, label="Uniform threshold", marker="o", s=80)
    plt.scatter(x + 0.08, thresholds, label="Equity-adjusted threshold", marker="s", s=80)
    plt.scatter(x, prior_vals, label="Illustrative subgroup prior", marker="^", s=80)
    plt.xticks(x, subgroups)
    plt.ylabel("Probability of clinically significant prostate cancer")
    plt.title("Illustrative subgroup-specific PSA/MRI decision thresholds")
    plt.legend()
    plt.tight_layout()
    plt.savefig(FIGDIR / "equity_psa_thresholds.png", dpi=300)
    plt.close()

def make_sepsis_pomdp_walkthrough():
    p0 = 0.25
    se1, sp1 = 0.85, 0.70
    se2, sp2 = 0.75, 0.90

    p1_pos = post_pos(p0, se1, sp1)
    p1_neg = post_neg(p0, se1, sp1)
    p2_pos = post_pos(p1_pos, se2, sp2)
    p2_neg = post_neg(p1_pos, se2, sp2)
    prob_test2_pos = se2 * p1_pos + (1 - sp2) * (1 - p1_pos)

    rows = [
        ["Initial assessment", p0, "Order Test 1", "No result yet", "Intermediate prior; information can change action"],
        ["After Test 1 positive", p1_pos, "Evaluate Test 2", "Near threshold", "Continue because Test 2 can separate treat from observe"],
        ["After Test 1 positive and Test 2 positive", p2_pos, "Stop testing", "High posterior", "Treat or escalate"],
        ["After Test 1 positive and Test 2 negative", p2_neg, "Stop testing", "Lower posterior", "Observe or pursue alternative diagnoses"],
        ["After Test 1 negative", p1_neg, "Stop biomarker sequence", "Low posterior", "Observe or reassess unless clinical status changes"],
    ]

```

```
    df = pd.DataFrame(
        rows,
        columns=["step", "posterior_belief", "action", "result_state
            ", "decision_implication"],
    )
    df.to_csv(OUT / "sepsis_pomdp_walkthrough.csv", index=False)

    fig, ax = plt.subplots(figsize=(12, 7))
    ax.set_xlim(0, 12)
    ax.set_ylim(0, 7)
    ax.axis("off")

    def box(x, y, w, h, text):
        r = FancyBboxPatch(
            (x, y), w, h,
            boxstyle="round,pad=0.02",
            linewidth=1.4,
            edgecolor="black",
            facecolor="white",
        )
        ax.add_patch(r)
        ax.text(x + w / 2, y + h / 2, text, ha="center", va="center"
            , fontsize=10, wrap=True)
        return (x, y, w, h)

    def arrow(b1, b2, label="", start="right", end="left"):
        x1, y1, w1, h1 = b1
        x2, y2, w2, h2 = b2

        if start == "right":
            p1 = (x1 + w1, y1 + h1 / 2)
        elif start == "bottom":
            p1 = (x1 + w1 / 2, y1)
        else:
            p1 = (x1 + w1 / 2, y1 + h1)

        if end == "left":
            p2 = (x2, y2 + h2 / 2)
        elif end == "top":
            p2 = (x2 + w2 / 2, y2 + h2)
        else:
            p2 = (x2 + w2 / 2, y2)

        ax.add_patch(
            FancyArrowPatch(
                p1,
                p2,
```

```
                arrowstyle="->",
                mutation_scale=14,
                linewidth=1.2,
                color="black",
            )
        )

        if label:
            ax.text(
                (p1[0] + p2[0]) / 2,
                (p1[1] + p2[1]) / 2 + 0.15,
                label,
                ha="center",
                va="bottom",
                fontsize=9,
            )

    b0 = box(0.3, 3.0, 2.1, 1.0, "Initial belief\nP(sepsis)=0.25")
    b1 = box(3.0, 3.0, 2.0, 1.0, "Order Test 1\nSe=0.85, Sp=0.70")
    bpos = box(5.8, 4.8, 2.3, 1.0, f"Test 1 positive\nPosterior={p1_pos:.3f}")
    bneg = box(5.8, 1.2, 2.3, 1.0, f"Test 1 negative\nPosterior={p1_neg:.3f}")
    b2 = box(8.7, 4.8, 2.0, 1.0, "Order Test 2\nSe=0.75, Sp=0.90")
    b2pos = box(9.6, 6.1, 2.1, 0.8, f"Test 2 positive\nPosterior={p2_pos:.3f}\nTreat/escalate")
    b2neg = box(9.6, 3.3, 2.1, 0.8, f"Test 2 negative\nPosterior={p2_neg:.3f}\nObserve/alternative")
    bstop = box(8.8, 1.2, 2.4, 1.0, "Stop biomarker sequence\nObserve/reassess")

    for a, b, l in [
        (b0, b1, ""),
        (b1, bpos, "positive"),
        (b1, bneg, "negative"),
        (bpos, b2, ""),
        (bneg, bstop, ""),
    ]:
        arrow(a, b, l)

    arrow(b2, b2pos, "positive", start="top", end="left")
    arrow(b2, b2neg, "negative", start="bottom", end="left")

    fig.tight_layout()
    fig.savefig(FIGDIR / "pomdp_sepsis_walkthrough.png", dpi=300, bbox_inches="tight")
    plt.close(fig)
```

```python

    return prob_test2_pos

def main():
    make_diagnostic_voi_figures()
    make_utility_and_equity_figures()
    make_sepsis_pomdp_walkthrough()

if __name__ == "__main__":
    main()
```

Listing 1: Python source code used to generate all manuscript figures.
